# Deriving and comparing healthy longevity distributions by gender and health prevalence measures: a statistical moments and maximum entropy approach

**DOI:** 10.1186/s12963-026-00470-9

**Published:** 2026-03-19

**Authors:** Rami Cosulich, Vanessa di Lego, Virginia Zarulli

**Affiliations:** 1 Department of Statistical Sciences, University of Padua, Padova, Italy; 2 Department of Demography, Cedeplar, Universidade Federal de Minas Gerais, Belo Horizonte, Brazil

**Keywords:** Healthy longevity distribution, Healthy lifespan variation, Outsurvival statistic, Hellinger distance, Health-survival paradox, Health inequalities.

## Abstract

**Background:**

The literature on healthy longevity has typically focused on average values (i.e., healthy life expectancy). Recent studies have started to expand this focus by investigating the whole healthy lifespan distribution, especially the standard deviation of healthy longevity, which captures inter-individual variation. Despite these advancements, research gaps remain on how distributions differ by health indicator and sex. This study aimed to compare healthy longevity distributions at age 60 between different health measures and sexes.

**Methods:**

We used data from the Survey of Health, Ageing and Retirement in Europe and the Human Mortality Database. A Markov chain model was used to estimate the first three statistical moments of healthy longevity distributions. The maximum entropy method was then applied to derive the full distributions. The healthy lifespan outsurvival statistic and the Hellinger distance were used to compare distributions between males and females.

**Results:**

For most health measures, the probabilities of health loss at younger ages were higher for males than for females, and females had a longer healthy life expectancy. Males had more dispersed distributions with a lower mode. For most health measures, healthy longevity distributions were negatively skewed, with a mode age (i.e., the age with the highest probability of health loss) higher than the healthy life expectancy age. The probability for a man to have a longer healthy lifespan than a female was below 50% for various health measures and was the lowest for living free of cardiovascular disease. In contrast, the probability for a man to live free of arthritis or rheumatism for longer than a female was above 50%. The most similar distributions between males and females were observed with life free of any chronic conditions and life with no more than one chronic condition.

**Conclusions:**

This study extended the scope of healthy longevity research by complementing a focus on the statistical moments with observations on the mode of the distributions and with formal comparisons based on the healthy lifespan outsurvival statistic and the Hellinger distance, which are applied for the first time in the healthy longevity field.

**Supplementary Information:**

The online version contains supplementary material available at 10.1186/s12963-026-00470-9.

## Background

Accurately capturing health inequality is one of the most pressing challenges in health research. The literature on healthy longevity has typically focused on average values (i.e., healthy life expectancy), highlighting fundamental differences between subpopulations distinguished by factors like socioeconomic status or gender. However, such average-based measures conceal how healthy lifespans are dispersed around the mean and how inequality unfolds within populations. In response to the limitations of relying solely on averages, recent studies have begun examining inter-individual variation in healthy longevity. Two studies [1, 2] addressed this topic globally, using Global Burden of Disease (GBD) data and the standard deviation as an indicator of variation. Moreover, one study assessed not only the standard deviation but also the skewness of healthy longevity distributions [3], using data from the Survey of Health, Ageing and Retirement in Europe (SHARE). Despite these latest efforts, unanswered questions remain on: (1) the shape of distributions of healthy lifespan, (2) how equally (or unequally) individuals lose their health, and (3) how the process of health loss differs by gender and by the measure of health prevalence used to define healthy longevity.

A focus on gender in healthy longevity research is particularly relevant because of the puzzling and persistent male–female health-survival paradox, which is the fact that women live longer but have poorer health than men [4, 5]. Despite extensive study and almost four decades of data confirming this gender gap, no single explanation fully accounts for why women live longer but have sicker lives. This leads to yet another major challenge in the field: the evaluation of healthy lifespan and gender differences is highly sensitive to the choice of health indicator. Estimates can vary widely depending on whether one uses self-rated health, functional limitations in activities of daily living, or chronic conditions - as highlighted in previous studies [6–11]. The fact that many indicators have been used depends both on the intrinsic multifaceted concept of health, which rightfully calls for many definitions to be used depending on the research interest, but also on the difficulty of operationalizing it univocally. An additional issue is that, contrary to incidence-based health definitions, definitions of health based on prevalence data score poorly at capturing the real progression of health loss of individuals over age, which is often made of transitions back and forth between the healthy and unhealthy status [12, 13]. Nevertheless, prevalence-based approaches remain the most used ones due to the abundance of data and the ease of measurement, compared to incidence-based analyses [14]: for the calculation of incidence by age, data should be longitudinal, it should be restricted to people who do not have the condition of interest at baseline, and should include information on the age at diagnosis. Moreover, the sample size typically becomes smaller when it is restricted to people followed up over time, for example due to some participants becoming unavailable or refusing to participate again (refreshment samples cannot be used as follow-up observations in incidence estimations, but can be used for prevalence estimates).

Without the presumption to address the substantive and normative aspects related to the ways health prevalence is operationalized in the demography of health, this study adopted a comparative approach to investigate healthy longevity distributions by gender using seven distinct measures of health prevalence, each representing a different aspect of health. These measures were defined specifically to minimize the inability of prevalence data to capture the dynamic transitions back and forth between healthy and unhealthy states, because they represent health statuses that are very difficult to fully recover from. Given that this paper focuses on chronic conditions, we use the term “health loss” to refer to a person reaching the end of their healthy lifespan, which can occur either by death or by transitioning to an unhealthy state (which is a permanent state). In addition, given our focus on gender differences, we prioritized the inclusion of conditions with known sex-specific impacts on health. With this approach, we can look at how the process of health loss unfolds and accumulates over age, differently for men and women, and for different measures of health.

We provide a full characterization of the healthy longevity distributions through the statistical moments (mean (healthy life expectancy), variance (inequality), and skewness (asymmetry)), which has been shown to provide a broader picture of health across populations, beyond an exclusive focus on healthy life expectancy [3, 12]. We report the standard deviation rather than the variance for ease of interpretability and because it is common practice in demography, and we call the standard deviation of healthy longevity (SDHL) “inter-individual variation in healthy longevity”, following previous work [1, 2]. We also present the full empirical distribution of health loss over the lifespan, derived with the maximum entropy method. Despite its high predictive power, applications of this method in demography so far have been limited to age at death distributions in the context of mortality forecasting [15]. For the first time, we have used it to model the health loss process, enabling us to examine the detailed shape of healthy lifespan distributions, including the distribution mode and the probabilities of losing health at specific ages. We show the evolution of distributions over time, and the differences between men and women across varying definitions of health. In particular, we make a formal comparison of gender differences through two measures that are applied for the first time in the healthy longevity field: the healthy lifespan outsurvival statistic ($\varphi {HL}$ statistic), expressed as the probability for a male to have a longer healthy lifespan compared to a female, and the Hellinger distance, used to measure the dissimilarity between the distributions of healthy years of life of men and women.

By moving beyond averages and analyzing the full distribution of healthy longevity, this study offers deeper insights into the dynamics of health inequality. It provides both a formal quantification of individual variation and a richer understanding of how health loss unfolds unequally between men and women across different dimensions of health.

## Methods

### The Markov chain with rewards model

We applied a Markov chain with rewards model [3] to estimate the mean, variance (reported as standard deviation for ease of interpretability), and skewness of healthy lifespan from prevalence data. The estimates are generated from two stochastic processes: the risk of mortality and the chance of developing health conditions, estimated by the prevalence of such conditions at each age. The model [3] accounts for both. The model requires age-specific mortality and health prevalence data, for which we used different health measures, outlined below.

### The maximum entropy method

From the first three moments of the distribution estimated with the Markov chain model, we derived the empirical probability distribution of the age at exit of healthy life (healthy lifespan) with the maximum entropy method [15]. This method makes the least-biased estimate possible when having incomplete information, by choosing a probability density function (PDF) that matches the known set of constraints, in this case the first three empirical moments of a distribution, and at the same time, maximizes the Shannon entropy to have the highest possible uncertainty in order to avoid introducing any additional hidden bias.

### Data: SHARE and HMD

Data on health prevalence come from various health measures included in the Survey of Health, Ageing and Retirement in Europe (SHARE), which is a study conducted approximately every two years, starting with wave 1, which was conducted in 2004 or in 2004/05 in most countries [16]. We used SHARE and easySHARE data from waves 2, 4, 5, 6, 7, 8, 9 [17–24] (details on the funding of SHARE are provided in the acknowledgments section). In most countries, wave 2 was conducted in 2006/07, wave 4 in 2011, wave 5 in 2013, wave 6 in 2015, wave 7 in 2017, wave 8 in 2019/2020, and wave 9 in 2021/22 [16]. We did not include wave 1 because it had no data on whether respondents had a dementia or Alzheimer’s diagnosis, which was used to define two cognitive measures used in this study. Moreover, we did not use wave 3 because it focused on the respondents’ life histories rather than using a regular questionnaire [16], so it had no relevant data. We did not use data from the SHARE Corona surveys because it did not include information on some of the conditions relevant to our analysis.

We combined SHARE’s health prevalence data with the annual probability of death for each specific age, sex, country and year from the Human Mortality Database (HMD) [25]. More details on this data merge are reported in Additional File 1, Supplementary Table S1.

SHARE has been conducted in 28 European countries and in Israel, but only some countries have participated in all waves [16]. We only included 9 countries that participated in all the SHARE waves outlined above and under the condition that they had HMD data for all the relevant years: Austria, Belgium, Czech Republic, Denmark, France, Italy, Spain, Sweden, Switzerland. Germany was not included because there was no HMD life table data for 2021 or 2022 [25], when SHARE wave 9 was conducted [16]. The Netherlands was not included because in waves 6 and 7, it did not participate in the regular SHARE waves but conducted a mixed mode experiment, which involved using an online survey or telephone interviewing instead of face-to-face interviews as the rest of the SHARE countries [16, 26, 27].

SHARE surveys people aged 50 and over but we restricted our analysis to people aged 60 and over because refreshment samples were missing for some country-wave combinations, and in some countries, this occurred for some consecutive waves [16, 28, 29]. This meant that people in their early- and mid-50s were missing or limited to younger partners. Due to sample size limitations, we grouped data with an open-ended interval for ages 90 and above.

### Health measures

Because our concern is with differences between women and men, gender-specific health conditions have been the focus in calculating health prevalence. The health measures taken from SHARE to calculate health prevalence are outlined in Table 1 (more details provided in Additional File 1, Supplementary Table S2), together with why they are specifically important for understanding gender differences in health and mortality.

### Data preparation

In our analysis, we applied the individual calibrated cross-sectional weights available within the SHARE dataset. These weights “may help reduce the potential selection bias generated by nonresponse errors” [16] (p.42) and enable inference from the responding sample to the target population [16]. Each weight takes into account the household design weight, the NUTS1 region (Nomenclature of Territorial Units for Statistics – Level 1), the gender and age class of the respondent [16]. Therefore, our analysis used a weighted number of respondents. For each wave, age and sex, we calculated health prevalence and a weighted average of the annual probability of death by pooling data across all countries (the weighted numbers of respondents for each combination of wave, age, sex and country were used as the weights for the calculation of the weighted average, see Supplementary Table S3 in Additional File 1 for details).

**Table 1:** Health measures: definitions and rationales for their inclusion

Health Measure	Definition	Rationale
Life free from major chronic conditions	Free from: high blood pressure, high cholesterol, heart problems, stroke or cerebral vascular disease, diabetes/high blood sugar, chronic lung disease, arthritis/rheumatism, Parkinson’s, Alzheimer’s/dementia	These conditions show significant gender disparities: women are more affected by arthritis and Alzheimer’s, while men have higher prevalence of lung disease and high blood sugar [30–33]
Life with no more than one chronic condition	Either no condition or only one from the list in the row above	Focuses on multimorbidity: it is linked to how long individuals remain relatively healthy before developing two or more chronic conditions
Life free from any cardiovascular disease (CVD)	Free from: heart problems, stroke or cerebral vascular disease	CVD significantly contributes to gender disparities in health outcomes: although men have higher prevalence and mortality at younger ages, women experience greater disability and death burden due to late diagnosis and less aggressive treatment [34, 35]
Life free from cardiovascular conditions/risk factors	Free from: high blood pressure, high cholesterol, heart problems, stroke or cerebral vascular disease	Includes key risk factors for CVDs to capture earlier stages of cardiovascular health deterioration
Life free from arthritis or rheumatism	Free from arthritis or rheumatism	Women are more likely than men to develop arthritis and live fewer years free from it; arthritis explains over 30% of the sex difference in ADLs, a key health expectancy measure [36]
Good cognitive health (general population comparison)	Scoring above − 1.5 SD from the mean on cognitive tests (time orientation, verbal fluency, memory), relative to all wave-person observations, and no dementia/Alzheimer’s diagnosis	It is a relevant indicator in relation to gender because women have a higher risk and earlier onset of Alzheimer’s than men [32, 37]
Good cognitive health (education-adjusted comparison)	Same as the measure above, but scores are evaluated relative to all wave-person observations with the same educational level	Accounts for educational disparities in cognitive test performance, making cognitive health comparisons more equitable

ADLs: activities of daily living; CVD: cardiovascular disease; SD: standard deviation

### From the Markov chain model to the maximum entropy method and the formal comparison of healthy longevity distributions through the healthy lifespan outsurvival statistic and the Hellinger distance

**Fig. 1:**
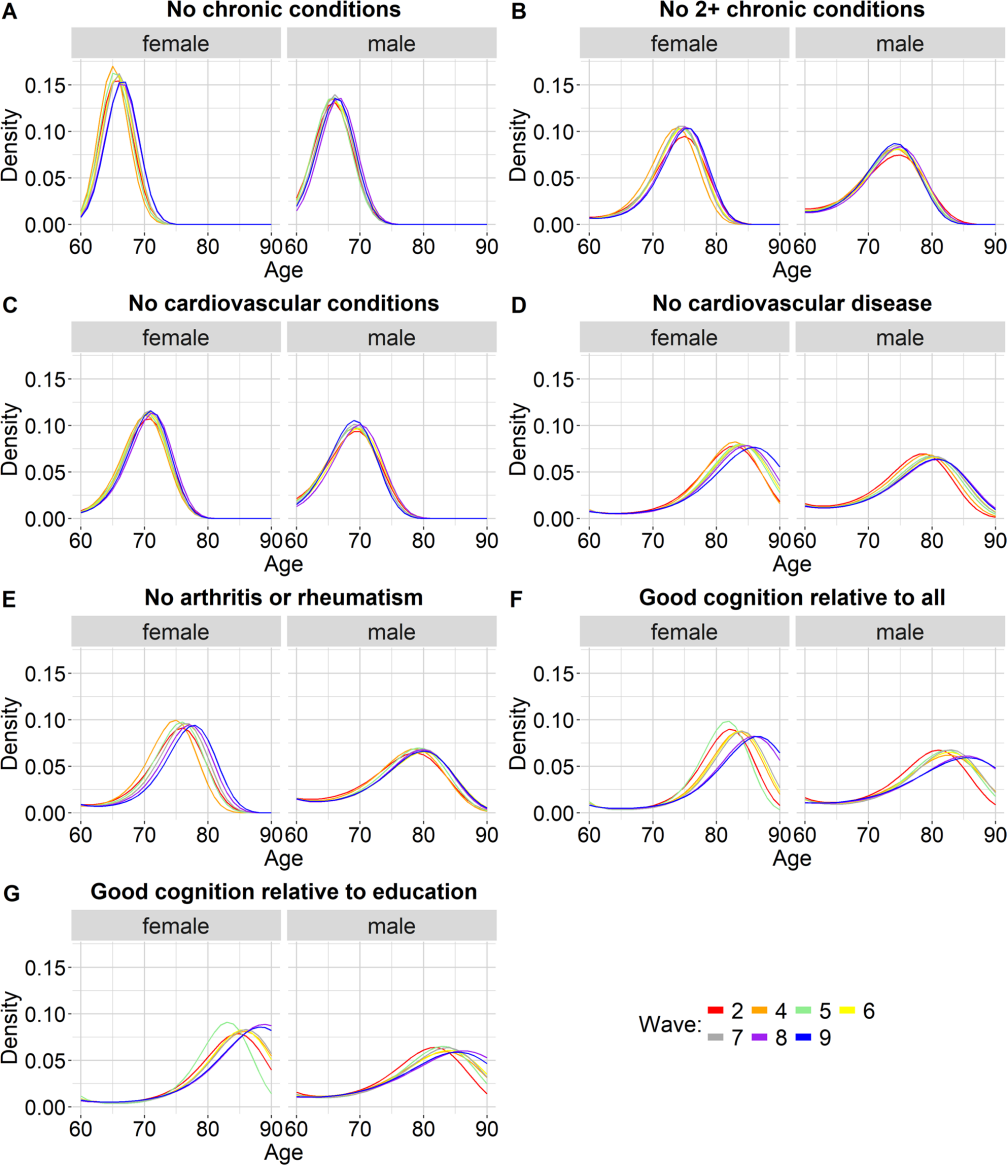
Probability density functions for different health measures: free from chronic conditions (panel A); no more than one chronic condition (panel B); free from cardiovascular conditions (panel C); free from cardiovascular disease (panel D); free from arthritis or rheumatism (panel E); good cognition relative to all (panel F); good cognition relative to education (panel G). Functions based only on point estimates to simplify the visual representation

Using the first three statistical moments of the healthy longevity distributions, calculated for each health measure and sex with the Markov chain model [3], we applied the maximum entropy method [15] to derive the full empirical distribution of health loss over the lifespan. In particular, this method estimated the probability of health loss at each age (the sum of all probabilities between age 60 and 90 was 1). Based on this, we treated the distributions as discrete, although we also present the continuous density functions (Fig. 1) to help visualize changes and similarities in the distributions over time.

We then compared the healthy longevity distributions between different sexes using both the healthy lifespan outsurvival statistic ($\varphi {HL}$ statistic) and the Hellinger distance. In studies on lifespan distributions, $\varphi$ is an outsurvival statistic that expresses “the probability that an individual from a population with lower life expectancy outlives an individual from another population with higher life expectancy” (p.2) [38]. In our study, $\varphi {HL}$ expresses the probability for a male to have a longer healthy lifespan than a female, when comparing randomly selected healthy men and women of age 60, assuming independence between the healthy lifespan of men and women (in agreement with the assumptions in [38]). The complementary value of $\varphi {HL}$, i.e., 1 - $\varphi { HL}$, refers to the probability for a female to have a longer healthy lifespan compared to a male. Note that when $\varphi {HL}$ is equal to 0.5, the probability for a man to have a longer healthy lifespan than a woman is exactly the same as the probability for a woman to have a longer healthy lifespan compared to a man.

The Hellinger distance measures the dissimilarity between distributions. It is closely related to the Bhattacharyya coefficient [39], which in turn, is a measure of overlap between different distributions [40]. The higher the Hellinger distance, the more dissimilar the distributions. The lower the Hellinger distance, the more similar the distributions. The Hellinger distance was prioritized over other measures such as Kullback-Leibler divergence because it is symmetric [41], meaning that the distance from distribution A to distribution B is the same as from B to A, so it is easier to use and interpret. Moreover, differently from Kullback-Leibler divergence, it is bounded [41] (typically between 0 and 1 [41], although an upper bound of 2 applies to the formula used in this study, outlined below).

We combined the use of $\varphi {HL}$ and the Hellinger distance as we believed this was the best way to tackle the analysis of the distributional differences. The $\varphi {HL}$ statistic formally relates two distributions by focusing on the part on which they overlap, while the Hellinger distance not only accounts for the overlap but is sensitive to distributional differences on the entire support. We were then able to get a complete picture of the differences between the distributions, not only capturing discrepancies or similarities in shape, but also computing a probabilistic indicator of relevant demographic significance (the $\varphi {HL}$ statistic).

### Calculation of $\varphi {HL}$

We used a discrete approximation of the main formula for φ from studies on longevity distributions [38, 42]. We applied it to healthy longevity (HL) through the formula presented in Eq. (1). The formula refers to $\varphi {HL}$ defined as the probability that an individual from population 1 has a longer healthy lifespan than an individual from population 2:

1 $$\begin{aligned} \varphi HL & \approx{\sum}_{x=60}^{w}({}_{n}{phloss}_{x}^{2}*{}_{n}{lhealth}_{x+n}^{1}) \\ & \quad +\frac{\sum_{x=60}^{w}({}_{n}{phloss}_{x}^{1}*{}_{n}{phloss}_{x}^{2})}{2} \end{aligned}$$

Where ${}_{n}{phloss}_{x}^{i}$ is the probability of health loss between age $x$ and $x+n$ in population $i$. $n$ is the width of the age group, so, when $n$ is 1, we are referring to single ages (for example, 70, 71, 72). ${}_{n}{lhealth}_{x+n}^{i}$ is the probability of being healthy until age $x+n$ for people in population $i$. For example, in our dataset focused on people who are healthy at 60, if $x$=70 and $n$=1, and if population 1 refers to males and population 2 to females, ${}_{1}{phloss}_{70}^{F}$ refers to the probability of losing health when aged 70 for females who are healthy at 60, and ${}_{1}{lhealth}_{70+1}^{M}$ refers to the probability of being healthy until age 71 for males who are healthy at 60. $w$ is the final age when anyone who is still healthy loses health. In our dataset, $w$ = 90, which is the age limit artificially imposed by our analysis due to data limitations.

### Calculation of the Hellinger distance

We calculated the Hellinger distance applying the formulas for discrete probability distributions from [43], shown in Eqs. (2) and (3).

2 $$HD=\sqrt{2*\sum_{x=60}^{w}{(\sqrt{{}_{n}{phloss}_{x}^{1}}-\sqrt{{}_{n}{phloss}_{x}^{2}})}^{2}}$$

3 $$=2\mathrm{*}\sqrt{1-\sum_{x=60}^{w}\sqrt{{}_{n}{phloss}_{x}^{1}\mathrm{*}{}_{n}{phloss}_{x}^{2}}}$$

where ${}_{n}{phloss}_{x}^{i}$ and $x$, $w,$ $n$, $i$ are defined as per Eq. (1). Note that the order of ${}_{n}{phloss}_{x}^{1}$ and ${}_{n}{phloss}_{x}^{2}$ is interchangeable in Eqs. (2) and (3).

After applying the formulas above, we validated the calculation using the R package Philentropy [44] to calculate the Hellinger distance

### Accounting for the sampling error around the prevalence point estimates

To incorporate sampling uncertainty around the prevalence point estimates, weighted non-parametric bootstrapping was used, applying the individual calibrated cross-sectional weights available within the SHARE dataset. More specifically, for each combination of health measure, wave, sex and age, weighted sampling with replacement was done 5,000 times, thus obtaining 5,000 prevalence values. The Markov chain model and the maximum entropy code were then run 5,000 times. 95% confidence intervals were obtained from the 5,000 sets of results. More specifically, the 2.5th and 97.5th percentiles of the results were the lower and upper limits of the confidence intervals.

### Sample size

For health measures 1 to 5 (i.e., the health measures that referred to chronic conditions rather than to the cognitive test scores), the unweighted sample per wave-sex-age combination ranged from a minimum of 22 males aged 89 in wave 2 to a maximum of 887 females aged 65 in wave 5. For the measures related to cognition, the sample was smaller, ranging from a minimum of 19 males aged 89 in wave 2 to a maximum of 765 females aged 66 and 765 females aged 67 in wave 6. One of the reasons for the more missing data for cognition is that time orientation questions were only asked to baseline respondents in waves 4 and 5, although the same questions were asked to both baseline and longitudinal respondents in the other waves.^1^ Wave 5 had a lower percentage of baseline respondents than wave 4, and so, for most ages, it had the lowest sample size for cognition. Moreover, anyone who responded to a SHARELIFE questionnaire in wave 7 (i.e., anyone who had not previously participated in wave 3 [16]) was not asked questions on time orientation and verbal fluency in wave 7. See Supplementary Figure S1 and Supplementary Tables S4-S7 in Additional File 1 for more details on the sample size.

### Final notes on the methods to help with the interpretation of the results

Before moving on to the results, it is worth reminding the reader that distributions of healthy longevity from age 60 apply to people who reach age 60 in a healthy state (defined differently depending on the health measure used). It may also help the reader to note that the distributions of healthy longevity can alternatively be interpreted as distributions of the age of health loss, defined as per the introduction section, i.e., the age at the end of healthy lifespan. The analysis is based on prevalence data and, therefore, does not allow for transitions back to a healthy state.

## Results

### Statistical moments of the healthy longevity distributions: comparison between different health measures

**Fig. 2:**
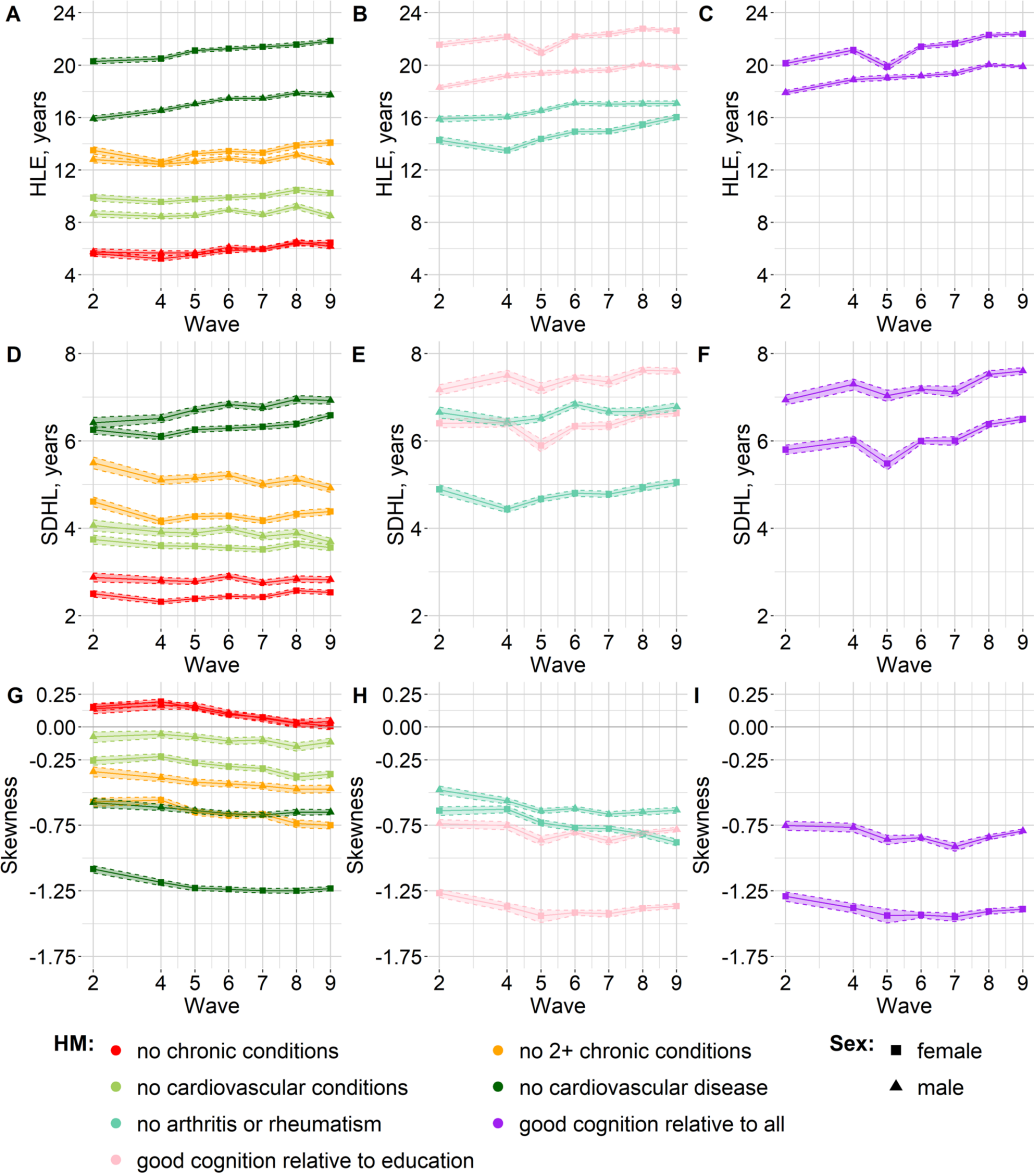
Statistical moments of healthy longevity distributions, namely, HLE (panels A, B, C), SDHL (panels D, E, F) and skewness (panels G, H, I), for different health measures: free from chronic conditions, no more than one chronic condition, free from cardiovascular conditions, free from cardiovascular disease (panels A, D, G); free from arthritis or rheumatism, good cognition relative to education (panels B, E, H), good cognition relative to all (panels C, F, I).HLE: healthy life expectancy (mean of the healthy longevity distribution); HM: health measure; SDHL: standard deviation of healthy longevity. Symbols (squares or triangles) connected by continuous line: point estimates. Dashed lines: 95% confidence intervals

For both males and females, and across all waves, life free of any chronic conditions was characterized by both the shortest healthy life expectancy (HLE) and the lowest standard deviation in healthy longevity (SDHL), i.e., the lowest inter-individual variation. Life free of any cardiovascular conditions had the second-lowest HLE and SDHL. Note that within each sex, SDHL was positively and very strongly correlated with HLE. Figure 1 shows the probability density functions of healthy longevity distributions from age 60, by wave, sex and health measure. Figure 2 shows the first three statistical moments (mean (i.e., HLE), standard deviation, and skewness) of the distributions. In Additional File 1, Supplementary Figure S2 shows the correlation between the statistical moments and Supplementary Table S8 outlines the definitions of correlation strength in the current work.

**Fig. 3:**
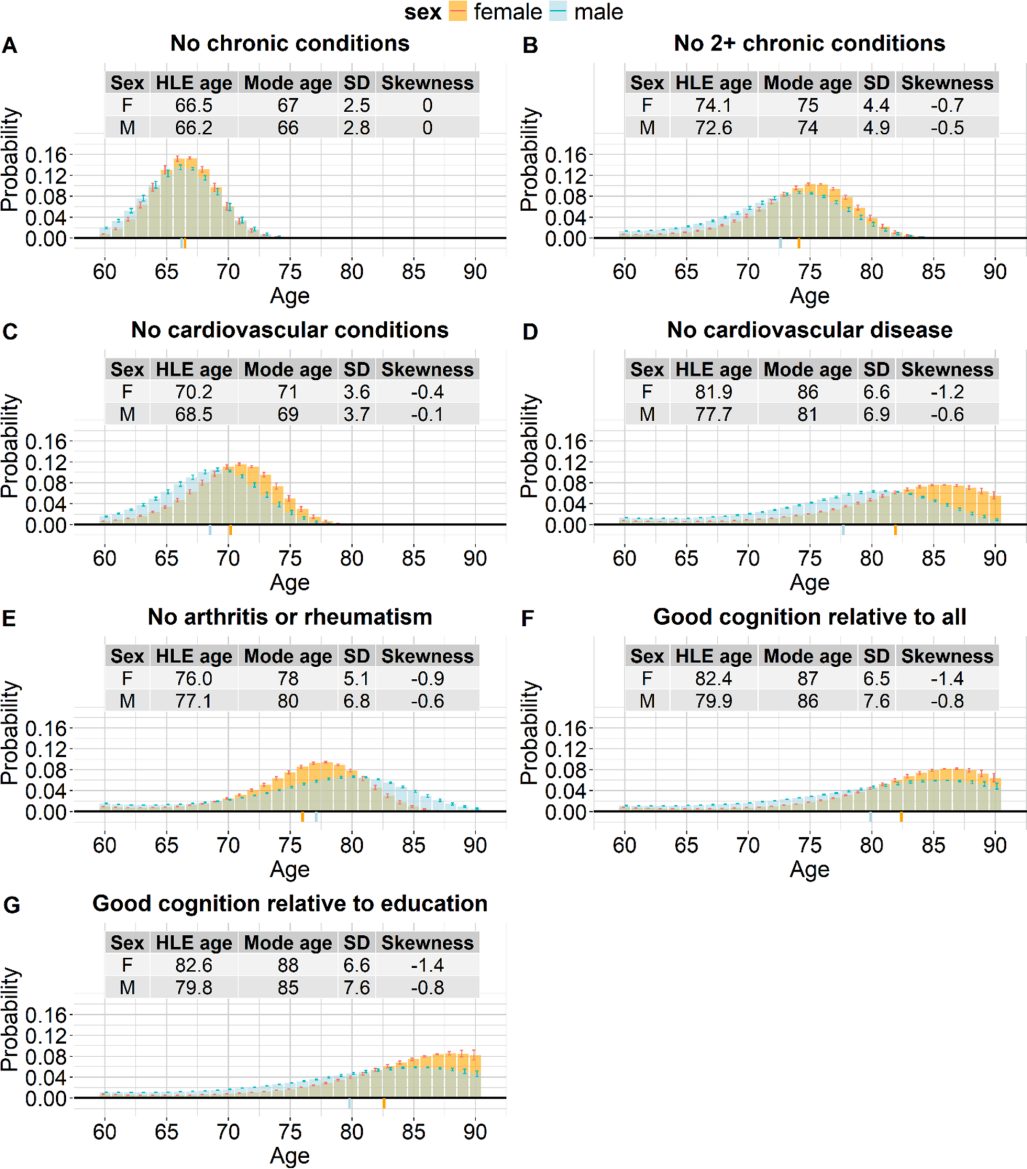
Age at the end of the healthy lifespan: probability mass functions, wave 9, for different health measures: free from chronic conditions (panel A); no more than one chronic condition (panel B); free from cardiovascular conditions (panel C); free from cardiovascular disease (panel D); free from arthritis or rheumatism (panel E); good cognition relative to all (panel F); good cognition relative to education (panel G).F: female; HLE: healthy life expectancy; M: male; SD: standard deviation. Wave 9 was conducted in 2021/22. It was selected for Fig. 3 because it was the latest available wave. The green colour indicates the overlap between the distributions for males and females. The coloured lines below the x axis show the HLE age for each sex. The HLE age was equal to 60 + HLE at age 60. The 95% CIs for the probabilities of health loss are shown, but for simplicity, the reported values are only point estimates

Figure 2 shows that for all health measures except “no chronic conditions”, skewness was negative across all waves, for both males and females. For each sex, the negative skewness values furthest from 0 were observed for the two “good cognition” measures. This meant that there was a minority that reached the end of the good-cognition-lifespan considerably earlier than the majority. Within each sex, skewness was negatively and very strongly correlated with HLE (see Additional File 1, Supplementary Figure S2): health measures with a higher HLE (such as the good cognition measures) tended to have more negatively skewed distributions. Consistently with this, the skewness values closest to 0 were observed for two measures with relatively low HLE: “no chronic conditions” and “no cardiovascular conditions”. Life free of any chronic conditions had positive skewness point estimates across all waves, although in some waves the 95% CI ranged from negative to positive. When negative skewness was relatively far from 0 (i.e., with the exception of life free of cardiovascular conditions), the negatively skewed distributions had a mean age of health loss lower than the mode age, i.e., the age with the highest probability of health loss. This held across all waves, for both males and females. For example, for a woman healthy at age 60, in wave 9, the mean age of experiencing either cardiovascular disease or death was 81.9 (95% CI: 81.7 to 82.0) but the age with the highest probability of experiencing either cardiovascular disease or death was age 86 (95% CI: 85 to 87) (the probability of losing health at this mode age rather than at other ages was 7.7%, 95% CI: 7.6% to 7.7%). For wave 9, the point estimates of the HLE age and mode age are visible in Fig. 3, which shows the probability mass functions for wave 9, by sex and health measure. Supplementary Figure S3 in Additional File 1 shows the mode age and the HLE age for all waves, by sex and health measure, with the 95% CIs.

### Statistical moments of the healthy longevity distributions: comparison between males and females

For most health measures, HLE was higher for females than for males, across all waves. A notable exception was that life expectancy free of arthritis or rheumatism was higher for males than for females, across all waves. Moreover, life expectancy free of any chronic conditions or with no more than one chronic condition was similar between males and females. For all health measures, the inter-individual variation in healthy longevity (as measured by SDHL) was higher for males than for females, across most waves (there were a couple of exceptions where the 95% CIs overlapped). For all health measures, except for “no chronic conditions”, distributions were more negatively skewed for females than for males.

### Statistical moments of the healthy longevity distributions: observations over time

For most health measures, there was no statistical moment with a consistently increasing or decreasing trend over time: there were both increases and decreases between waves (see Fig. 2 and Supplementary Figures S4-S5 in Additional File 1). The only notable exception was life expectancy free of cardiovascular disease for females: the point estimate always increased from wave to wave. Comparing wave 9 to wave 2, the point estimate of HLE increased between these two waves, for all health measures (females) or most health measures (males). For both males and females, distributions with negative skewness became more negatively skewed in wave 9 compared to wave 2 (see Fig. 2 and Supplementary Figure S6 in Additional File 1).

For the good-cognition longevity distributions for females, there was noticeable inter-wave variation when comparing wave 5 to the other waves (see Figs. 1 and 2). This may be linked to the inter-wave variations in eligibility criteria for some cognition questions. As mentioned in the methods section, for most ages, wave 5 had the lowest sample size for cognition due to these eligibility criteria.

### Probabilities of health loss at specific ages and at modal age

For most health measures, the probabilities of health loss at younger ages were higher for males than for females. However, close to the modes of the healthy longevity distributions, probabilities of health loss were higher for females than for males, and at the oldest ages, probabilities of health loss were either higher for females or similar between sexes (see Fig. 3; note that for each sex, the sum of all probabilities across all ages is equal to 1). The only exception to this was life free of arthritis or rheumatism, which corresponded to higher probabilities of health loss for males than for females at older ages. Within the same health measure and within the same wave, the probability of health loss at the mode age was always higher for females than for males. Figure 3 shows this for wave 9. For example, in wave 9, for poor cognition relative to all other person-wave observations, the peak probability of health loss was 0.082 (95% CI 0.082 to 0.083) for females (at age 87, 95% CI: 86 to 87) and 0.059 (95% CI: 0.059 to 0.060) for males (at age 86, 95% CI: 85 to 87). Supplementary Figure S7 in Additional File 1 shows the probability of health loss at mode age for all waves, by health measure and sex. The higher mode for females was consistent with the lower dispersion of the female distributions (which was measured by SDHL). Supplementary Figure S8 in Additional File 1 shows the relationship between the probability of health loss at mode age and SDHL.

Supplementary Figures S9-S10 in Additional File 1 show that the probability mass functions before the COVID-19 pandemic (in wave 7) were very similar to those for wave 9, during the pandemic.

### Results from $\varphi HL$ and Hellinger distance

The $\varphi HL$ statistic indicated that across all waves, men had a probability above 50% of living without arthritis or rheumatism for longer than females. Across all waves, for life free of cardiovascular disease, life free of cardiovascular conditions and life with good cognition, men had a probability below 50% of having a longer healthy lifespan than females (there was only one exception when the 95% CI of a good cognition measure crossed the 50% threshold in wave 5). $\varphi HL$ was the lowest for the health measure “no cardiovascular disease” across all waves. In most waves, $\varphi HL$ was closest to 0.5 for “no chronic conditions” and “no 2 + chronic conditions”, with the 95% CIs crossing 0.5 in some waves.

**Fig. 4:**
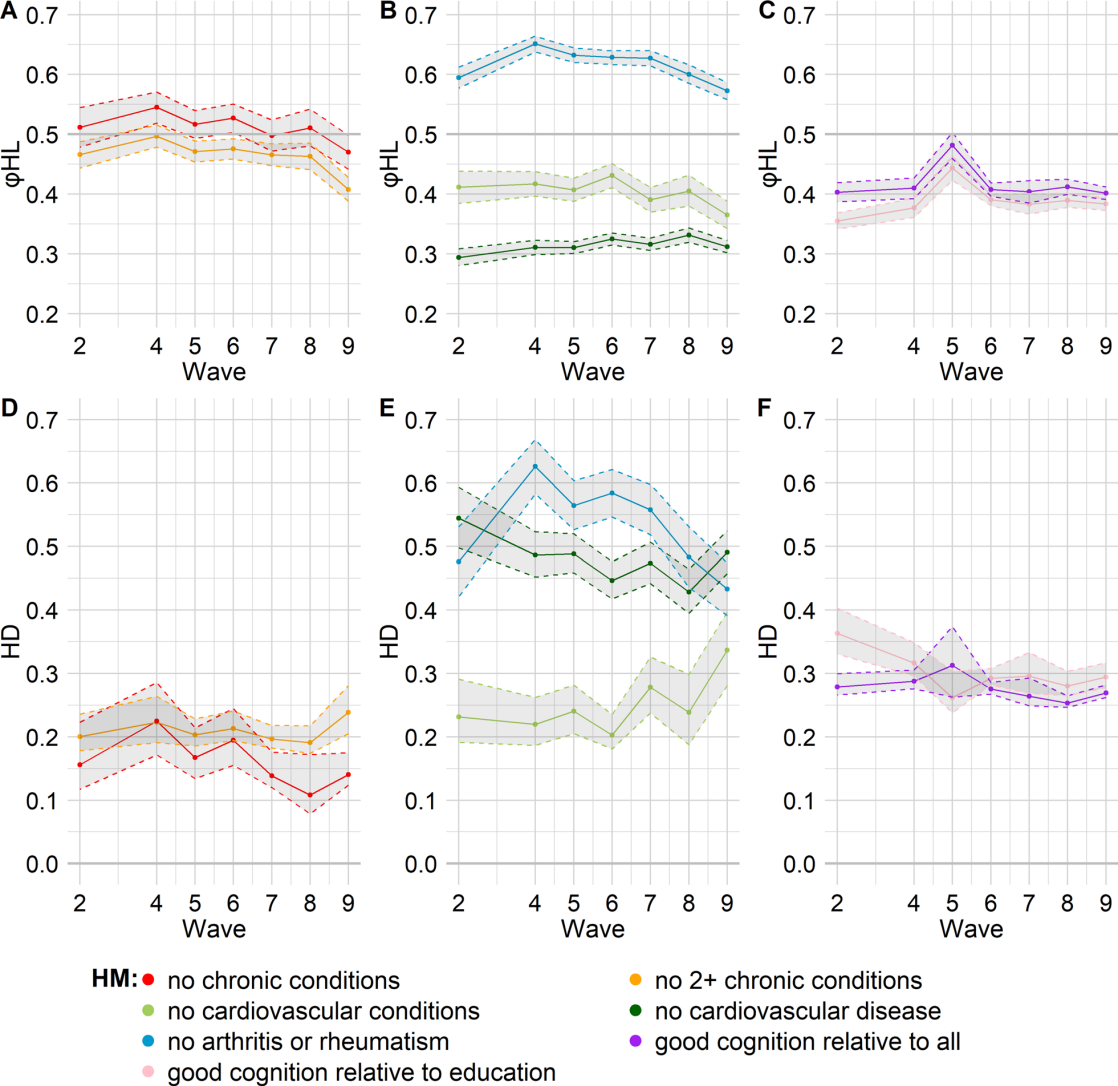
$\varphi { HL}$ (panels A, B, C) and Hellinger distance (panels D, E, F) over time for different health measures: free from chronic conditions, no more than one chronic condition (panels A, D); free from cardiovascular conditions, free from cardiovascular disease, free from arthritis or rheumatism (panels B, E); good cognition relative to all, good cognition relative to education (panels C, F).HD: Hellinger distance; HM: health measure. The $\varphi { HL}$ statistic expresses the probability for males to have a longer healthy lifespan than females. The Hellinger distance compares the healthy longevity distributions for males and females (the higher the Hellinger distance, the more dissimilar the distributions)

Figure 4 shows $\varphi HL$ and the Hellinger distance in different waves. The $\varphi HL$ statistic in wave 9 for cardiovascular disease indicated that if we randomly selected a man and a woman, both without cardiovascular disease at age 60, the man would have a probability of 31% (95% CI: 30% to 32%) of living without cardiovascular disease for a longer time compared to the woman. For arthritis and rheumatism, the $\varphi HL$ statistic in wave 9 indicated that if we randomly selected a man and a woman, both without arthritis or rheumatism at age 60, the man would have a probability of 57% (95% CI: 56% to 59%) of living without arthritis or rheumatism for longer than the woman. The Hellinger distance was the highest for “no arthritis or rheumatism” and for “no cardiovascular disease”, thus indicating the highest dissimilarity between the distributions of males and females when it comes to these health measures. Supplementary Figures S11-S12 in Additional File 1 show the point estimates of $\varphi HL$ and of the Hellinger distance next to the probability mass functions for wave 2 and wave 9. Supplementary Figure S13 in Additional File 1 shows the complementary value of $\varphi HL$ (1- $\varphi HL$), i.e., the probability of females to have a longer healthy lifespan compared to males, next to the probability mass functions for wave 9.

There was a strong and positive correlation between the Hellinger distance and the distance of $\varphi HL$ from 0.5 (based on point estimates, the Pearson’s correlation coefficient was 0.79; see Additional File 1, Supplementary Figure S14 for details).

## Discussion

This study compared healthy longevity distributions at age 60 between sexes and between different health measures, focusing on the first three statistical moments, the mode and the probabilities of losing health at specific ages. Moreover, the $\varphi HL$ statistic and the Hellinger distance were used to make formal comparisons between the male and female distributions.

Lifespan free of any chronic conditions corresponded to the lowest HLE, the lowest inter-individual variation and the only measure with positive or null skewness values. For most health measures, healthy longevity distributions for females had higher HLE, lower SDHL, higher mode and more negative skewness compared to the distributions for males, across all waves. An exception was “no arthritis or rheumatism”, with higher HLE for males than for females. This was the only health measure for which $\varphi HL$ consistently indicated that men had a probability above 50% of having a longer healthy lifespan than females. Moreover, for two comprehensive health definitions, i.e., “no chronic conditions” and “no 2 + chronic conditions”, there was little difference in HLE between males and females, and $\varphi HL$ was the closest to 50% for these two health measures, with the 95% CIs crossing 50% in some waves. The distributions that differed the most between males and females were for life free of arthritis and rheumatism and life free of cardiovascular disease, as indicated by $\varphi HL$ and the Hellinger distance.

To better understand the results, both the differences in mortality and in health prevalence should be considered. Once data had been pooled across all countries, as expected, the annual probability of death was higher for men than for women of the same age, across all waves (Supplementary Figure S15 in Additional File 1 shows this for wave 2 and wave 9). The prevalence of cardiovascular disease was either similar between males and females or higher for males, depending on age and wave (Supplementary Figure S16 in Additional File 1 shows health prevalence for wave 9). The combination of these factors resulted in the lowest $\varphi HL$ for life free of cardiovascular disease. Conversely, there was often a higher prevalence of arthritis or rheumatism among women than among men of the same age, which offset the higher male annual probability of death, leading to $\varphi HL$ above 50% for life free of these conditions. For life free of any chronic conditions or life with no more than one chronic condition, prevalence was similar between males and females. However, in various wave-age combinations, the point estimates of prevalence of any chronic conditions or 2 + chronic conditions were higher for females. When this was combined with the probabilities of death, the distributions of healthy longevity were similar between sexes. Note that the similar prevalence between males and females was somewhat unexpected because a previous study based on SHARE data had found higher odds of comorbidity among females compared to males [4]. However, the set of chronic conditions included in the current study only partly overlapped with the set of conditions included in that previous study.

To relate the findings from this study to the female-male health-survival paradox mentioned in the background section, note that despite the longer life expectancy of females, lifespan free of any chronic conditions was similar between males and females, and lifespan free of arthritis or rheumatism tended to be longer for males. For the other health measures with higher HLE for females, and with better prospects for females based on the healthy lifespan outsurvival statistic, the results are not necessarily in contrast to the health-survival paradox: further research should compare males and females not only in relation to the healthy lifespan, but also considering the distributions of unhealthy lifespan and the percentage of lifespan spent in good health. Indeed, Eurostat reports that in the EU, healthy life expectancy at birth in 2023 was higher for women than for men (63.3 vs. 62.8 years) but the percentage of life expectancy spent in good health was lower for women than for men (75.4% vs. 79.8%) [52]. These estimates are based on the Global Activity Limitation Instrument, where people are asked to what extent they have been limited in usual activities because of a health problem [53]. Future research could investigate this further using the measures selected for the current study and focusing on people who are still healthy at age 60. The present work found more negative skewness in female healthy longevity distributions, which contributes to understanding the female–male health-survival paradox: this pattern indicates that although most women experience relatively long healthy lifespans, there remains a subgroup experiencing substantially shorter healthy lifespans, resulting in an asymmetric distribution. Combined with women’s longer overall survival, this implies that women may spend more years in poor health despite having higher or similar healthy life expectancy. In contrast, the greater variability and less pronounced skewness among men reflect a wider dispersion of health trajectories. These distributional differences highlight that the paradox cannot be fully understood through healthy life expectancy alone, but requires considering the shape of the full healthy longevity distribution.

The finding that SDHL was higher for males than for females across all health measures and waves was consistent with the findings in [3], which also used SHARE data but defined healthy longevity differently: either as having no limitations in activities of daily living or based on hand grip strength. In both cases, the SDHL was higher for males than for females at age 55 and 75. In contrast, there were different findings relating to the comparison of SDHL between males and females in two publications based on data from the Global Burden of Disease (GBD) study on 204 countries, from the year 1990 to 2019 [1, 2]. In the GBD data, healthy lifespan was measured based on both years of life lost due to premature mortality and years lived with disability, which were calculated by applying disability weights to a variety of health conditions [54]. One study [1] found that at age 65, SDHL was higher for females than for males, on average. Similarly, the other study [2] found that SDHL at age 65 was higher for females than males in most country-year observations. The opposite findings in studies using SHARE vs. GBD data could be due to the specificities of the GBD definition of healthy longevity.

This study showed that, except for negative skewness values relatively close to 0, negative skewness was reflected in a modal age above the HLE age. At least for some health measures (in particular, those with relatively high probabilities of health loss at age 90), the finding of negative skewness may have been influenced by the artificial limit of age 90 imposed by the analysis: this meant that health loss at very old ages could not be captured by the distributions. Further research with bigger sample sizes could use higher age limits and could attempt to validate the skewness findings versus observational incidence-based studies.

Although the present work did not focus on disability-free life expectancy, some of the health measures that we included are related to disability: in particular, previous research found that self-reported arthritis was positively associated with ADL disability, IADL disability and mobility disability [55]. Moreover, cardiovascular disease can also lead to disability. On the one hand, men had a probability above 50% of living for more years than women without arthritis or rheumatism, but on the other hand, the probability was below 50% when it came to cardiovascular disease, across all waves (see Fig. 4). One study found no statistically significant differences between sexes in the age gradients of disability incidence in Northern, Central and Eastern Europe in their study focused on people aged 55–89, with disability defined as having any difficulty with ADLs, based on data from the Gateway to Global Aging. They found statistically significant differences in Southern Europe, where higher disability incidence in women was observed in the older age groups [56]. Further work could investigate how these results relate to the incidence of different chronic conditions by age, sex and region.

Focusing on the inter-wave differences of healthy longevity distributions, there were various waves with unique characteristics, which could have potentially led to distinct results for these waves, but the only wave that stood out visually was wave 5, for the cognition measures for females. This wave had restricted eligibility criteria for the cognition questions and the lowest sample size. Other peculiarities of specific waves were as follows: narrower eligibility criteria for the cognition questions were implemented not only in wave 5, but also in waves 4 and 7, as mentioned in the methods section; the questions around arthritis and rheumatism were different in wave 2 and 4 compared to the other waves (see Supplementary Table S2 in Additional File 1 for more details); wave 8 was interrupted prematurely due to the COVID-19 public health emergency [57]. By the time it was interrupted, around 70% of the expected longitudinal interviews and around 50% of the expected refreshment interviews had been completed, although this varied by country [57]. Moreover, for our study, mortality in wave 8 was based on 2019 and 2020, while for wave 9 it was based on 2021 and 2022. Therefore, some Covid-related mortality was incorporated into the healthy longevity estimations. Surprisingly, despite all these aspects, none of these waves stood out visually in the plots created for the current study.

This work has strengths and limitations. One strength is that, to our knowledge, this is the first study to apply the maximum entropy method to derive the full empirical distribution of health loss over the lifespan, and to compare the distributions of healthy longevity between different health measures. We also provide, for the first time, a formalization of the comparison between male and female healthy longevity distributions.

An important limitation is the use of prevalence-based health measures instead of incidence ones. The method assumes that not only death but also the unhealthy state is an absorbing state and does not allow for transitions back and forth between healthy and unhealthy. Because of this, we excluded from the analysis widely-used health measures that do not necessarily capture chronic health issues, such as self-rated health or being free from activity limitations, and we focused on conditions that it is very difficult to recover from, in order to minimize the problem of the possible transition back to full health.

Another limitation is that an average annual probability of death was used in the Markov chain model. However, in practice, there would be different probabilities of death for healthy and non-healthy people.

Moreover, our application of the maximum entropy method seems to underestimate the probabilities of health loss at older ages (towards the right tail of the distributions), and consequently, the probabilities of health loss at some younger ages are likely to be overestimated. See Supplementary Table S9 in Additional File 1 for details on the validation checks regarding this issue. Some deviation from empirical data is to be expected, especially because our application of the maximum entropy method only used the first three statistical moments. While the use of additional moments such as kurtosis would further improve accuracy, a previous study on age-at-death distributions showed that the predictive power with three moments was very effective, achieving a coverage of 93%, compared to only 80% achieved with only the first two moments [15]. Another reason for limiting our analysis to the use of the first three moments was that an article had been published on the application of the Markov chain model for the estimation of these moments [3]. See Supplementary Figures S17 and S18 in Additional File 1 for the probability density functions estimated using two and four moments. The aforementioned study on age-at-death distributions pointed out that more than four moments might be required to accurately capture multi-modal distributions [15]. However, given the selected chronic health measures, we expected healthy longevity from age 60 to follow a unimodal distribution. This expectation was based on the unimodality of distributions of age at death and age at onset of morbidity for older people: one study using data from the Human Mortality Database from 41 countries from 1751 to 2019 showed that at older ages, the distribution of ages at death was unimodal [58]. Moreover, another study, using data from the Global Burden of Diseases, Injuries, and Risk Factors Study 2019, presented incidence by age of various noncommunicable diseases, showing that, at older ages, there was a single peak [59]. For example, for some conditions (such as ischemic heart disease, stroke and intracerebral hemorrhage, Alzheimer’s disease, and chronic obstructive pulmonary disease), there was an increase in incidence with age that continued into the oldest age groups; for other conditions (such as Parkinson’s disease and atrial fibrillation), incidence increased with age but at the oldest ages it declined or levelled off [59]. In contrast, bimodal distributions may be expected for healthy longevity from birth because age-at-death distributions from birth are usually bimodal with both an infant and an adult mortality peak [60]. Future research should focus on further developing the methods to derive healthy longevity distributions so that potential multi-modal distributions can also be captured.

An additional limitation is that $\varphi HL$ compares randomly-paired individuals, assuming independence between the two populations, however, in practice, some of these individuals would be husbands and wives or cohabiting, thus influencing each other’s health [61–64]: for people participating from the first wave of SHARE, all household members born in 1954 or earlier were eligible for a SHARE interview. For the new countries entering the survey later and for the refreshment samples, there was only one selected respondent per household, but current partners living in the same household were also interviewed [16].

Furthermore, it should be noted that the health prevalence data are based on self-reporting of doctor’s diagnoses. The age-specific prevalence used to estimate the healthy longevity distributions may be linked to frequency of attendance at health care services, which may vary by sex. For example, a literature review on attendance at general health checks (including screening for cardiovascular disease) found that women had higher rates of attendance than men [65]. In turn, more frequent attendance could lead to earlier diagnosis.

This study is the first of its kind and opens the way to a variety of research avenues. For example, future work would need to compare healthy longevity distributions not only between sexes but also between different educational levels and other socio-economic and demographic variables. Bergeron-Boucher and colleagues point out that “males with a university degree or who are married have a higher chance of outliving females, in particular females with a lower education level and who are single” ([38], p. 6). Further research could also study coupled men and women. However, this kind of study could be done only on the SHARE countries (or other similar survey data) that have performed record linkage with the mortality registries, to use the correct probabilities of death, which refer to the subpopulations of individuals connected by the fact of being in a stable relationship. Additionally, future work could investigate healthy longevity distributions for older ages, specific countries or wider geographical regions.

## Conclusions

The maximum entropy method was useful to extend the focus of healthy longevity research beyond the statistical moments of distributions, namely by investigating the mode and by formalizing the comparisons between males and females through the $\varphi HL$ statistic and the Hellinger distance. This study has applied these measures for the first time in the healthy longevity field. As expected, the choice of health measure affected the comparison between sexes, but some findings were persistent across all health measures: males had higher inter-individual variation than females and females had a higher distribution mode. Males and females differed the least in their distributions when all chronic conditions were considered together; they differed the most in life free of cardiovascular disease and life free of arthritis or rheumatism.

## Supplementary Information

Below is the link to the electronic supplementary material.

Supplementary Material 1: See Additional File 1 (uploaded as file: “Healthy longevity distributions SHARE Additional File 1 2026.02.19 clean Zotero unlinked.docx”). This file includes Supplementary Tables S1-S9 and Supplementary Figures S1-S18.

## Data Availability

The datasets analysed during the current study [17–25] are available from the SHARE website (see [https://share-eric.eu/data/](https:/share-eric.eu/data) and see DOIs of references [17–24]) and from the HMD website (see [https://www.mortality.org/](https:/www.mortality.org) and see reference [25]) . The R code is available on 10.5281/zenodo.18958539

## Footnotes

1 This was observed during the data analysis. Also see the SHARE questionnaires for the filters applied in each wave [45–51].

## Abbreviations

ADL: Activity of daily living
CI: Confidence interval
CVD: Cardiovascular disease
HLE: Healthy life expectancy
HL: Healthy longevity or health loss
HD: Hellinger distance
HMD: Human mortality database
IADL: Instrumental activity of daily living
NUTS1: Nomenclature of territorial units for statistics–level 1
PDF: Probability density function
SDHL: Standard deviation of healthy longevity
SHARE: Survey of health, ageing and retirement in europe

## References

[1] Permanyer I, Villavicencio F, Trias-Llimós S. Healthy lifespan inequality: morbidity compression from a global perspective. Eur J Epidemiol. 2023;38:511–21. 10.1007/s10654-023-00989-3. 37027116 10.1007/s10654-023-00989-3 PMC10080172

[2] Zarulli V, Caswell H. Longer healthy life, but for how many? A stochastic analysis of healthy lifespan inequality. Ann Oper Res [Internet]. 2024. 10.1007/s10479-024-06203-1. [cited 2025 Jan 17]. 10.1007/s10479-024-06203-1 PMC13457341 42582296

[3] Caswell H, Zarulli V. Matrix methods in health demography: a new approach to the stochastic analysis of healthy longevity and DALYs. Popul Health Metr. 2018;16:8. 10.1186/s12963-018-0165-5. 29879982 10.1186/s12963-018-0165-5 PMC5992869

[4] Ahrenfeldt LJ, Möller S, Thinggaard M, Christensen K, Lindahl-Jacobsen R. Sex Differences in Comorbidity and Frailty in Europe. Int J Public Health. 2019;64:1025–36. 10.1007/s00038-019-01270-9. 31236603 10.1007/s00038-019-01270-9 PMC7237816

[5] Oksuzyan A, Juel K, Vaupel JW, Christensen K. Men: good health and high mortality. Sex differences in health and aging. Aging Clin Exp Res. 2008;20:91–102. 10.1007/BF03324754. 18431075 10.1007/bf03324754 PMC3629373

[6] Di Lego V, Di Giulio P, Luy M. Gender Differences in Healthy and Unhealthy Life Expectancy. In: Jagger C, Crimmins EM, Saito Y, De Carvalho Yokota RT, Van Oyen H, Robine J-M, editors. Int Handb Health Expect [Internet]. Cham: Springer International Publishing; 2020. pp. 151–72. [cited 2025 July 21]. 10.1007/978-3-030-37668-0_11.

[7] Di Lego V, Nepomuceno MR, Turra CM. Gender gaps in healthy life expectancy as indicators of inequality for disability and chronic disease: cross-sectional evidence from 24 countries, years 2014–2019. Br Med J Publishing Group. 2025. 10.1136/bmjopen-2024-096968. [cited 2025 Nov 21]. 10.1136/bmjopen-2024-096968 PMC12636968 41263830

[8] Di Lego V, Sauerberg M. The Sensitivity of the Healthy Life Years Indicator: Approaches for Dealing with Age-Specific Prevalence Data. Comp Popul Stud [Internet]. 2023 [cited 2025 Oct 31];48. 10.12765/CPoS-2023-06

[9] Reinwarth AC, Wicke FS, Rückert KK, Schattenberg JM, Tüscher O, Wild PS, et al. Change of self-rated physical health predicts mortality in aging individuals: results of a population-based cohort study. Arch Public Health. 2024;82:130. 10.1186/s13690-024-01363-9. 39180092 10.1186/s13690-024-01363-9 PMC11342511

[10] Van Oyen H, Cox B, Jagger C, Cambois E, Nusselder W, Gilles C, et al. Gender gaps in life expectancy and expected years with activity limitations at age 50 in the European Union: associations with macro-level structural indicators. Eur J Ageing. 2010;7:229–37. 10.1007/s10433-010-0172-2. 28798631 10.1007/s10433-010-0172-2 PMC5547327

[11] Van Oyen H, Nusselder W, Jagger C, Kolip P, Cambois E, Robine J-M. Gender differences in healthy life years within the EU: an exploration of the health–survival paradox. Int J Public Health. 2013;58:143–55. 10.1007/s00038-012-0361-1. 22618297 10.1007/s00038-012-0361-1 PMC3557379

[12] Caswell H, van Daalen S. Healthy longevity from incidence-based models: More kinds of health than stars in the sky. Demogr Res. 2021;45:397–452. 10.4054/DemRes.2021.45.13.

[13] Murray CJ, Salomon JA, Mathers C. A critical examination of summary measures of population health. Bull World Health Organ. 2000;78:981–94. 10994282 PMC2560826

[14] Schroeder SA. Incidence, prevalence, and hybrid approaches to calculating disability-adjusted life years. Popul Health Metr. 2012;10:19. 10.1186/1478-7954-10-19. 22967055 10.1186/1478-7954-10-19 PMC3462112

[15] Pascariu MD, Lenart A, Canudas-Romo V. The maximum entropy mortality model: forecasting mortality using statistical moments. Scand Actuar J Taylor Francis. 2019;2019:661–85. 10.1080/03461238.2019.1596974.

[16] SHARE. Survey of Health, Ageing and Retirement in Europe (SHARE) Release Guide 9.0.0 [Internet]. 2024 [cited 2025 Jan 15]. https://share-eric.eu/fileadmin/user_upload/Release_Guides/SHARE_release_guide_9-0-0.pdf. Accessed 15 Jan 2025.

[17] SHARE-ERIC. Survey of Health, Ageing and Retirement in Europe (SHARE) Wave 2. Release version: 9.0.0. SHARE-ERIC. Data set. 2024. 10.6103/SHARE.w2.900

[18] SHARE-ERIC. Survey of Health, Ageing and Retirement in Europe (SHARE) Wave 4. Release version: 9.0.0. SHARE-ERIC. Data set. 2024. 10.6103/SHARE.w4.900

[19] SHARE-ERIC. Survey of Health, Ageing and Retirement in Europe (SHARE) Wave 5. Release version: 9.0.0. SHARE-ERIC. Data set. 2024. 10.6103/SHARE.w5.900

[20] SHARE-ERIC. Survey of Health, Ageing and Retirement in Europe (SHARE) Wave 6. Release version: 9.0.0. SHARE-ERIC. Data set. 2024. 10.6103/SHARE.w6.900

[21] SHARE-ERIC. Survey of Health, Ageing and Retirement in Europe (SHARE) Wave 7. Release version: 9.0.0. SHARE-ERIC. Data set. 2024. 10.6103/SHARE.w7.900

[22] SHARE-ERIC, SHARE-ERIC. Survey of Health, Ageing and Retirement in Europe (SHARE) Wave 8. Release version: 9.0.0. Data set. 2024. 10.6103/SHARE.w8.900.

[23] SHARE-ERIC. Survey of Health, Ageing and Retirement in Europe (SHARE) Wave 9. Release version: 9.0.0. SHARE-ERIC. Data set. 2024. 10.6103/SHARE.w9.900

[24] SHARE-ERIC, easySHARE. Release version: 9.0.0. SHARE-ERIC. Dataset. [Internet]. 2024 [cited 2024 Aug 16]. 10.6103/SHARE.easy.900

[25] Human Mortality Database (HMD). Period Data. Life Tables - Male. Life Tables - Female [Internet]. 2024 [cited 2024 Dec 18]. https://www.mortality.org/Data/ZippedDataFiles. Accessed 18 Dec 2024.

[26] SHARE. Dutch mixed mode experiment W6 [Internet]. 2024 [cited 2026 Feb 9]. https://share-eric.eu/data/data-set-details/dutch-mixed-mode-experiment-w6-1. Accessed 9 Feb 2026.

[27] SHARE. Dutch mixed mode experiment W7 [Internet]. 2024 [cited 2026 Feb 9]. https://share-eric.eu/data/data-set-details/dutch-mixed-mode-experiment-w6. Accessed 9 Feb 2026.

[28] Bergmann M, Börsch-Supan A, editors. SHARE wave 8 methodology: collecting cross-national survey data in times of COVID-19 [Internet]. Munich: MEA, Max Planck Institute for Social Law and Social Policy. 2021 [cited 2025 Jan 17]. https://share-eric.eu/fileadmin/user_upload/Bilder_Newsredaktion/SHARE_Methodenband_WEB__1_.pdf. Accessed 17 Jan 2025.

[29] Bergmann M, Kneip T, De Luca G, Scherpenzeel A. Survey participation in the Survey of Health, Ageing and Retirement in Europe (SHARE), wave 1–7. [Internet]. 2019 [cited 2025 Mar 15]. https://share-eric.eu/fileadmin/user_upload/SHARE_Working_Paper/WP_Series_41_2019_Bergmann_et_al.pdf. Accessed 15 Mar 2025.

[30] Crimmins EM, Kim JK, Hagedorn AL. With and Without Disease: Women Experience More of Both. J Women Aging [Internet]. Taylor & Francis Group; 2002 [cited 2025 Mar 12]; 10.1300/J074v14n01_04 10.1300/J074v14n01_04 12537279

[31] Namavari N, Jokar M, Ghodsian A, Jahromi HK, Rahmanian V. Menopausal state and rheumatoid arthritis: a systematic review and meta-analysis. BMC Rheumatol. 2024;8:48. 10.1186/s41927-024-00418-2. 39350181 10.1186/s41927-024-00418-2 PMC11441135

[32] Nebel RA, Aggarwal NT, Barnes LL, Gallagher A, Goldstein JM, Kantarci K, et al. Understanding the impact of sex and gender in Alzheimer’s disease: A call to action. Alzheimers Dement J Alzheimers Assoc. 2018;14:1171–83. 10.1016/j.jalz.2018.04.008. 10.1016/j.jalz.2018.04.008 PMC6400070 29907423

[33] Ntritsos G, Franek J, Belbasis L, Christou MA, Markozannes G, Altman P, et al. Gender-specific estimates of COPD prevalence: a systematic review and meta-analysis. Int J Chron Obstruct Pulmon Dis. 2018;13:1507–14. 10.2147/COPD.S146390. 29785100 10.2147/COPD.S146390 PMC5953270

[34] Townsend N, Nichols M, Scarborough P, Rayner M. Cardiovascular disease in Europe — epidemiological update 2015. Eur Heart J. 2015;36:2696–705. 10.1093/eurheartj/ehv428. 26306399 10.1093/eurheartj/ehv428

[35] Vogel B, Acevedo M, Appelman Y, Bairey Merz CN, Chieffo A, Figtree GA, et al. The Lancet women and cardiovascular disease Commission: reducing the global burden by 2030. Lancet Lond Engl. 2021;397:2385–438. 10.1016/S0140-6736(21)00684-X. 10.1016/S0140-6736(21)00684-X 34010613

[36] Whitson HE, Landerman LR, Newman AB, Fried LP, Pieper CF, Cohen HJ. Chronic medical conditions and the sex-based disparity in disability: the Cardiovascular Health Study. J Gerontol Biol Sci Med Sci. 2010;65:1325–31. 10.1093/gerona/glq139. 10.1093/gerona/glq139 PMC2990264 20675619

[37] Beam CR, Kaneshiro C, Jang JY, Reynolds CA, Pedersen NL, Gatz M. Differences Between Women and Men in Incidence Rates of Dementia and Alzheimer’s Disease. J Alzheimers Dis JAD. 2018;64:1077–83. 10.3233/JAD-180141. 30010124 10.3233/JAD-180141 PMC6226313

[38] Bergeron-Boucher M-P, Alvarez J-A, Kashnitsky I, Zarulli V. Probability of males to outlive females: an international comparison from 1751 to 2020. BMJ Open [Internet]. British Medical Journal Publishing Group; 2022 [cited 2025 Mar 3];12:e059964. 10.1136/bmjopen-2021-059964. 10.1136/bmjopen-2021-059964 PMC9472123 35918112

[39] Nilsson N, Håkansson B, Ortiz-Catalan M. Classification complexity in myoelectric pattern recognition. J Neuroeng Rehabil. 2017;14:68. 10.1186/s12984-017-0283-5. 28693533 10.1186/s12984-017-0283-5 PMC5504674

[40] Guillerme T, Cooper N. Effects of missing data on topological inference using a Total Evidence approach. Mol Phylogenet Evol. 2016;94:146–58. 10.1016/j.ympev.2015.08.023. 26335040 10.1016/j.ympev.2015.08.023

[41] Ding R, Mullhaupt A. Empirical Squared Hellinger Distance Estimator and Generalizations to a Family of α-Divergence Estimators. Entropy. 2023;25:612. 10.3390/e25040612. 37190400 10.3390/e25040612 PMC10137612

[42] Vaupel JW, Bergeron-Boucher M-P, Kashnitsky I. Outsurvival as a measure of the inequality of lifespans between two populations. Demogr Res Max-Planck-Gesellschaft zur Foerderung der Wissenschaften. 2021;44:853–64. https://www.jstor.org/stable/27032937.

[43] Cha SH. Comprehensive survey on distance/similarity measures between probability density functions. Int J Math models methods Appl Sci. 2007;4:300–7.

[44] Drost HG. Philentropy: information theory and distance quantification with R. J Open Source Softw. 2018. 10.21105/joss.00765.

[45] SHARE. SHARE w2 questionnaire version 2.7 2006-09-21 [Internet]. 2006 [cited 2025 Mar 15]. https://share-eric.eu/fileadmin/user_upload/Questionnaires/Q-Wave_2/w2_en_capi_main-Generic.pdf. Accessed 15 Mar 2025.

[46] SHARE. Main questionnaire. Wave 4. [Internet]. n.d. [cited 2025 Mar 15]. https://share-eric.eu/fileadmin/user_upload/Questionnaires/Q-Wave_4/w4_en_capi_main-Generic.pdf. Accessed 15 Mar 2025.

[47] SHARE. Main questionnaire & end of life questionnaire. Wave 5 [Internet]. n.d. [cited 2025 Mar 15]. https://share-eric.eu/fileadmin/user_upload/Questionnaires/Q-Wave_5/w5_en_capi_main-Generic.pdf. Accessed 15 Mar 2025.

[48] SHARE. Main questionnaire & end of life questionnaire. Wave 6 [Internet]. n.d. [cited 2025 Mar 15]. https://share-eric.eu/fileadmin/user_upload/Questionnaires/Q-Wave_6/w6_en_capi_main-Generic.pdf. Accessed 15 Mar 2025.

[49] SHARE. Main questionnaire. Wave 7. [Internet]. n.d. [cited 2025 Mar 15]. https://share-eric.eu/fileadmin/user_upload/Questionnaires/Q-Wave_7/w7_en_capi_main-Generic.pdf. Accessed 15 Mar 2025.

[50] SHARE. Main questionnaire. Wave 8. [Internet]. n.d. [cited 2025 Mar 15]. https://share-eric.eu/fileadmin/user_upload/Questionnaires/Q-Wave_8/paperverstion_en_GB_8_2_5b.pdf. Accessed 15 Mar 2025.

[51] SHARE. Main questionnaire. Wave 9. [Internet]. n.d. [cited 2025 Mar 15]. https://share-eric.eu/fileadmin/user_upload/Questionnaires/Q-Wave_9/paperversion_en_GB_9_2_2a.pdf. Accessed 15 Mar 2025.

[52] Eurostat. Healthy life years statistics [Internet]. 2025 [cited 2026 Feb 11]. https://ec.europa.eu/eurostat/statistics-explained/index.php?title=Healthy_life_years_statistics#:~:text=Highlights,for%20women%20and%20men%2C%20respectively. Accessed 11 Feb 2026.

[53] Eurostat. Reference metadata. Healthy life years by sex (from 2004 onwards) (hlth_hlye). 2020. https://ec.europa.eu/eurostat/cache/metadata/en/hlth_hlye_esms.htm

[54] Institute for Health Metrics and Evaluation (IHME). Glossary of Terms [Internet]. 2016 [cited 2025 Mar 13]. https://www.healthdata.org/sites/default/files/files/policy_report/GBD/2016/IHME_GBD2015_Report_Glossary-of-terms_2016.pdf. Accessed 13 Mar 2025.

[55] Liu X, Huang Y, Fu J, Mohedaner M, Danzengzhuoga, Yang G, et al. Associations of arthritis with functional disability and depressive symptoms in general US adults: NHANES 1988–1994 and 1999–2018. AGING Med. 2024;7:705–16. 10.1002/agm2.12379. 10.1002/agm2.12379 PMC11702379 39777093

[56] Lee J, Meijer E, Phillips D, Hu P. Disability Incidence Rates for Men and Women in 23 Countries: Evidence on Health Effects of Gender Inequality. J Gerontol Ser A. 2021;76:328–38. 10.1093/gerona/glaa288. 10.1093/gerona/glaa288 PMC7812438 33216874

[57] Scherpenzeel A, Axt K, Bergmann M, Douhou S, Oepen A, Sand G, et al. Collecting survey data among the 50 + population during the COVID-19 outbreak: The Survey of Health, Ageing and Retirement in Europe (SHARE). Surv Res Methods. 2020;14:217–21. 10.18148/srm/2020.v14i2.7738.

[58] Beltrán-Sánchez H, Fernandez O. The evolving statistical signature of human mortality: skewness, kurtosis and lifespan disparity. R Soc Open Sci. 2026;13:251307. 10.1098/rsos.251307.

[59] Le Couteur DG, Thillainadesan J. What Is an Aging-Related Disease? An Epidemiological Perspective. J Gerontol Ser A. 2022;77:2168–74. 10.1093/gerona/glac039. 10.1093/gerona/glac039 PMC9678203 35167685

[60] Vázquez-Castillo P, Bergeron-Boucher M-P, Missov T. Longevity à la mode: A discretized derivative tests method for accurate estimation of the adult modal age at death. Demogr Res. 2024;50:325–46. 10.4054/DemRes.2024.50.11.

[61] Rasulo D, Christensen K, Tomassini C. The Influence of Social Relations on Mortality in Later Life: A Study on Elderly Danish Twins. Gerontologist. 2005;45:601–8. 10.1093/geront/45.5.601. 16199394 10.1093/geront/45.5.601

[62] Perelli-Harris B, Hoherz S, Addo F, Lappegård T, Evans A, Sassler S, et al. Do Marriage and Cohabitation Provide Benefits to Health in Mid-Life? The Role of Childhood Selection Mechanisms and Partnership Characteristics Across Countries. Popul Res Policy Rev. 2018;37:703–28. 10.1007/s11113-018-9467-3. 30546176 10.1007/s11113-018-9467-3 PMC6267248

[63] Staehelin K, Schindler C, Spoerri A, Stutz EZ. Group for the SNCS. Marital status, living arrangement and mortality: does the association vary by gender? J Epidemiol Community Health. Volume 66. BMJ Publishing Group Ltd; 2012. pp. e22–22. 10.1136/jech.2010.128397. 10.1136/jech.2010.128397 22012962

[64] Kiecolt-Glaser JK, Wilson SJ, Lovesick. How Couples’ Relationships Influence Health. Annu Rev Clin Psychol. 2017;13:421–43. 10.1146/annurev-clinpsy-032816-045111. 28301763 10.1146/annurev-clinpsy-032816-045111 PMC5549103

[65] Dryden R, Williams B, McCowan C, Themessl-Huber M. What do we know about who does and does not attend general health checks? Findings from a narrative scoping review. BMC Public Health. 2012;12:723. 10.1186/1471-2458-12-723. 22938046 10.1186/1471-2458-12-723 PMC3491052

[66] Börsch-Supan A, Brandt M, Hunkler C, Kneip T, Korbmacher J, Malter F, et al. Data resource profile: the Survey of Health, Ageing and Retirement in Europe (SHARE). Int J Epidemiol. 2013;42:992–1001. 10.1093/ije/dyt088. 23778574 10.1093/ije/dyt088 PMC3780997

[67] Börsch-Supan A, Brugiavini A, Jürges H, Kapteyn A, Mackenbach J, Siegrist J, et al. editors. First result from the Survey of Health, Ageing and Retirement in Europe (2004–2007). Starting the longitudinal dimension. Mannheim: Mannheim Research Institute for the Economics of Aging (MEA). [Internet]. Mannheim: Mannheim Research Institute for the Economics of Aging (MEA); 2008 [cited 2025 Nov 27]. https://share-eric.eu/fileadmin/user_upload/First_Results_Books/FRB2_all_chapters.pdf. Accessed 27 Nov 2025.

[68] Börsch-Supan A, Brandt M, Hank K, Schröder M, editors. The Individual and the Welfare State: Life Histories in Europe [Internet]. Berlin Heidelberg: Springer-Verlag; 2011 [cited 2025 Nov 27]. 10.1007/978-3-642-17472-8. Accessed 27 Nov 2025.

[69] Schröder M, editor. Retrospective data collection in the Survey of Health, Ageing and Retirement in Europe. SHARELIFE methodology. Mannheim: Mannheim Research Institute for the Economics of Aging (MEA). [Internet]. 2011 [cited 2025 Nov 27]. https://share-eric.eu/fileadmin/user_upload/Methodology_Volumes/FRB-Methodology_feb2011_color-1.pdf. Accessed 27 Nov 2025.

[70] Börsch-Supan A, Brandt M, Litwin H, Weber G, editors. Active ageing and solidarity between generations in Europe: First results from SHARE after the economic crisis [Internet]. Göttingen: De Gruyter; 2013. [cited 2025 Nov 27]. 10.1515/9783110295467.

[71] Malter F, Börsch-Supan A, editors. SHARE Wave 4: Innovations & Methodology [Internet]. Munich: MEA, Max Planck Institute for Social Law and Social Policy. 2013 [cited 2025 Nov 27]. https://share-eric.eu/fileadmin/user_upload/Methodology_Volumes/Method_FRB_FINAL_Wave4.pdf. Accessed 27 Nov 2025.

[72] Börsch-Supan A, Thorsten K, Litwin H, Myck M, Weber G, editors. Ageing in Europe - Supporting Policies for an Inclusive Society [Internet]. Berlin: De Gruyter; 2015. [cited 2025 Nov 27]. 10.1515/9783110444414.

[73] Malter F, Börsch-Supan A, editors. SHARE wave 5: innovations and methodology [Internet]. Munich: MEA, Max Planck Institute for Social Law and Social Policy. 2015 [cited 2025 Nov 27]. https://share-eric.eu/fileadmin/user_upload/Methodology_Volumes/Method_vol5_31March2015.pdf. Accessed 27 Nov 2025.

[74] Malter F, Börsch-Supan A, editors. SHARE wave 6: panel innovations and collecting dried blood spots. [Internet]. Munich: Munich Center for the Economics of Aging (MEA); 2017 [cited 2025 Jan 17]. https://share-eric.eu/fileadmin/user_upload/Methodology_Volumes/201804_SHARE-WAVE-6_MFRB.pdf. Accessed 17 Jan 2025.

[75] Bergmann M, Scherpenzeel A, Börsch-Supan A, editors. SHARE wave 7 methodology: panel innovations and life histories. [Internet]. Munich: Munich Center for the Economics of Aging (MEA); 2019 [cited 2025 Jan 17]. https://share-eric.eu/fileadmin/user_upload/Methodology_Volumes/SHARE_Methodenband_A4_WEB_Wave7MFRB.pdf. Accessed 17 Jan 2025.

[76] Bergmann M, Wagner M, Börsch-Supan A, editors. SHARE wave 9 methodology: from the SHARE Corona survey 2 to the SHARE main wave interview. [Internet]. Munich: SHARE-ERIC; 2024 [cited 2025 Jan 17]. https://share-eric.eu/fileadmin/user_upload/Methodology_Volumes/SHARE_Methodenband_WEB_Wave9.pdf. Accessed 17 Jan 2025.

[77] Gruber S, Hunkler C, Stuck S. Generating easySHARE: guidelines, structure, content and programming. SHARE Working Paper Series. 2014. https://share-eric.eu/fileadmin/user_upload/SHARE_Working_Paper/SHARE_WP_Series_17_2014.pdf

