# Supplementary material for: Deriving and comparing healthy longevity distributions by gender and health prevalence measures: a statistical moments and maximum entropy approach
Source: Popul Health Metr. 2026 Mar 19;24:18. doi: 10.1186/s12963-026-00470-9 (PMC13001290; doi:10.1186/s12963-026-00470-9)
Supplement: Supplementary file 1 — Supplementary Material 1: See Additional File 1 (uploaded as file: “Healthy longevity distributions SHARE Additional File 1 2026.02.19 clean Zotero unlinked.docx”). This file includes Supplementary Tables S1-S9 and Supplementary Figures S1-S18. [file 12963_2026_470_MOESM1_ESM.docx]

# **Additional File 1**

**Deriving and Comparing Healthy Longevity Distributions by Gender and Health Prevalence Measures: A Statistical Moments and Maximum Entropy Approach**

**Supplementary Table S1. Details on the merge between SHARE and HMD.**

| **Details on the merge** | Some SHARE waves were run across different calendar years, so we calculated a weighted average of the annual probability of death (qx) for each country across different calendar years. The weights were the proportions of people aged 50 or over interviewed in each year of the wave for a specific country. For example, in Austria, wave 2 was conducted in 2006/07. 26.6% of people aged 50 and over were interviewed in 2006 and 73.4% in 2007. For a male of age 60, qx was 0.01069 in 2006 and 0.01026 in 2007. The weighted average of qx for males of age 60 in Austria in wave 2 was calculated as follows: 0.01069*0.266+0.01026*0.734 = 0.01037 |
| --- | --- |
| **Exceptions** | Exceptions to the above were Spain, Italy and the Czech Republic in wave 9. These countries had SHARE data for wave 9 (2021/2022) but only HMD data for 2021, with no HMD data for 2022, so mortality from 2021 was assigned for wave 9. |

**Table notes.** Abbreviations: HMD: Human Mortality Database; SHARE: Survey of Health, Ageing and Retirement in Europe.

**Supplementary Table S2. Details on the health measures**

| **Chronic condition outlined in the main text** | **More details (citations are from the SHARE questionnaires)** [1–7] |
| --- | --- |
| Heart problems | The questionnaire asked about: “A heart attack including myocardial infarction or coronary thrombosis or any other heart problem including congestive heart failure”. |
| Chronic lung disease | The questionnaire asked about: “chronic lung disease such as chronic bronchitis or emphysema”. |
| Arthritis or rheumatism | In waves 2 and 4, the questionnaire asked about “arthritis, including osteoarthritis, or rheumatism”. In waves 5 to 9, two separate questions were asked, one about “rheumatoid arthritis” and one about “osteoarthritis, or other rheumatism”. |
| Dementia or Alzheimer’s diagnosis | The questionnaire asked about “Alzheimer’s disease, dementia, organic brain syndrome, senility or any other serious memory impairment”. |
| Cognitive measures | The cognitive tests taken into account were:   - Immediate recall (how many words can be recalled after listening to a list of 10 words); - Delayed recall (how many words can be recalled from a list of 10 words after some time); - Orientation in time (four questions about the current year, month, day of the month and day of the week); - Verbal fluency (the person is asked to name as many animals as they can in 60 seconds).   For each person, the z-score for each test was calculated. For “poor cognition relative to all”, this z-score was relative to all person-wave observations across all countries and waves. For “poor cognition relative to education”, the z-score was relative to person-wave observations with the same educational level (across all countries and waves).  For each person, the z-scores from different tests were averaged together. Then, this average was standardized into its own z-score. (A similar approach was used in [8]). This was used to assess whether a person’s cognitive score was at least 1.5 SDs below the mean. The same threshold of 1.5 SDs below the mean had been used in [9] to define mild cognitive impairment.  People with a cognitive score at least 1.5 SDs below the mean or with a dementia or Alzheimer’s diagnosis were classified as with poor cognition. People with a cognitive score above the threshold and with no dementia or Alzheimer’s diagnosis were classified as with good cognition. If people had missing information on a dementia diagnosis, their cognition was classified as NA, even if they had information on the cognitive test scores. However, if they had a dementia diagnosis but no information on the cognitive test scores, they were classified as with poor cognition, because it was considered possible that the missing information on the cognitive test scores could be due to the presence of dementia in some cases (we observed that the proportion with missing information was higher among those with dementia). Additionally, we assigned NA for cognition to longitudinal respondents in waves 4 and 5, and SHARELIFE respondents in wave 7, because there was missing information on some cognitive tests due to the survey design.  For poor cognition relative to education, SHARE includes the International Standard Classification of Education (ISCED) 1997. We used the original ISCED categories for the most part, but we grouped these categories together: “still in school” and “other” were grouped together. Moreover, we included different missing codes (“don’t know/refusal” and “no information”) into a single category. |

**Table notes.** We included the chronic conditions for which there was data across all relevant SHARE waves. For chronic conditions, the questionnaire differed depending on whether the person was a baseline or longitudinal respondent: For baseline respondents, the question was "Has a doctor ever told you that you had…" whereas for longitudinal respondents the question was "Do you currently have…". [10] (p.21).

**Supplementary Table S3. Weighted average of qx when pooling data across countries.**

| **Calculation** | After calculating a weighted average of qx for each country-wave combination (see Supplementary Table S1 for details), data were pooled across all countries by wave, sex, age. In order to do this, a weighted average (WA) of qx was calculated with this formula:  WA (qx) _wave_w, sex_s, age_a_  =  (qx _wave_w, sex_s, age_a, country_1_  * weighted_N_wave_w, sex_s, age_a, country_1_ +  qx _wave_w, sex_s, age_a, country_2_  * weighted_N_wave_w, sex_s, age_a, country_2_ +  …. +  qx _wave_w, sex_s, age_a, country_n_  * weighted_N_wave_w, sex_s, age_a, country_n_)  /  (weighted_N_wave_w, sex_s, age_a, country_1_ +  weighted_N_wave_w, sex_s, age_a, country_2_ +  …. +  weighted_N_wave_w, sex_s, age_a, country_n_) |
| --- | --- |
| **Notes** | Weighted_N is the number of people after applying the cross-sectional individual calibrated weights, and it refers to people with available data on the health variables of interest, so it changed depending on whether the focus was on chronic conditions or on poor cognition. |
| **Example** | In wave 2, the weighted N of women aged 60 who had data on chronic conditions was 54766 for Austria and 73802 for Belgium. So when qx for women aged 60 in wave 2 across all countries was calculated, Austria had a weight of 54766 and Belgium of 73802. This is consistent with the calculation of health prevalence across all countries, where Belgium also had a bigger weight than Austria. |

**
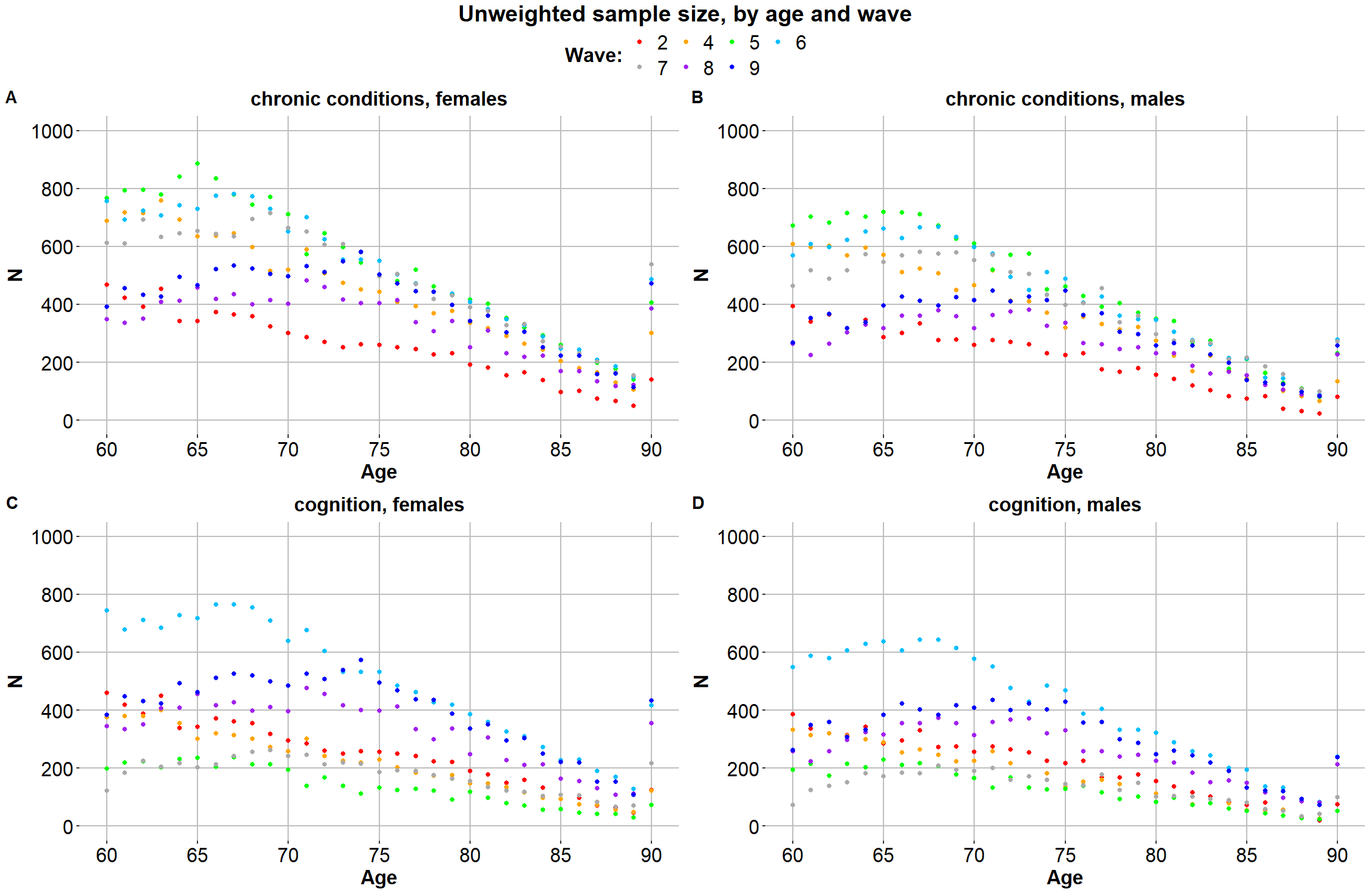
**

**Supplementary Figure S1. Unweighted sample size.**

**Supplementary Table S4. Unweighted sample size by wave, people with information on chronic conditions.**

| **Wave** | **Sample size** |
| --- | --- |
| 2 | 14240 |
| 4 | 24734 |
| 5 | 30731 |
| 6 | 29516 |
| 7 | 27507 |
| 8 | 18323 |
| 9 | 22228 |
| **TOT** | 167279 |

**Supplementary Table S5. Unweighted sample size by wave, people with available data to define poor cognition.**

| **Wave** | **Sample size** |
| --- | --- |
| 2 | 13969 |
| 4 | 12085 |
| 5 | 8201 |
| 6 | 28309 |
| 7 | 9531 |
| 8 | 17949 |
| 9 | 21715 |
| **TOT** | 111759 |

**Supplementary Table S6. Data availability on cognition, wave 4 and wave 5.**

| **Wave** | **Baseline respondents** | | **No information on whether baseline or longitudinal respondents** | | **Longitudinal respondents (excluded from the analyses on cognition due to missing information on time orientation)** | **Tot** |
| --- | --- | --- | --- | --- | --- | --- |
|  | **With data to define poor cognition** | **No data to define poor cognition** | **With data to define poor cognition** | **No data to define poor cognition** |  |  |
| 4 | 12085 (48.9%) | 312 (1.3%) | 0 | 21 | 12316 (49.8%) | 24734 (100%) |
| 5 | 8199  (26.7%) | 304 (1.0%) | 2 | 16 | 22210 (72.3%) | 30731 (100%) |

**Table notes.** Number of people (% in brackets). The two people without information on whether they were baseline/longitudinal respondents had information on their cognitive test scores as well as on dementia.

**Supplementary Table S7. Data availability on cognition, wave 7.**

| **Did not respond to SHARELIFE and so had regular wave 7 questionnaire** | | **Responded to SHARELIFE and so had condensed wave 7 questionnaire (excluded from the analyses on cognition due to missing information on time orientation and verbal fluency)** | **Tot** |
| --- | --- | --- | --- |
| **Had relevant data to define poor cognition** | **Had no relevant data to define poor cognition** |  |  |
| 9531 | 240 | 17736 | 27507 |

**Table notes.** The table shows the number of people.

**
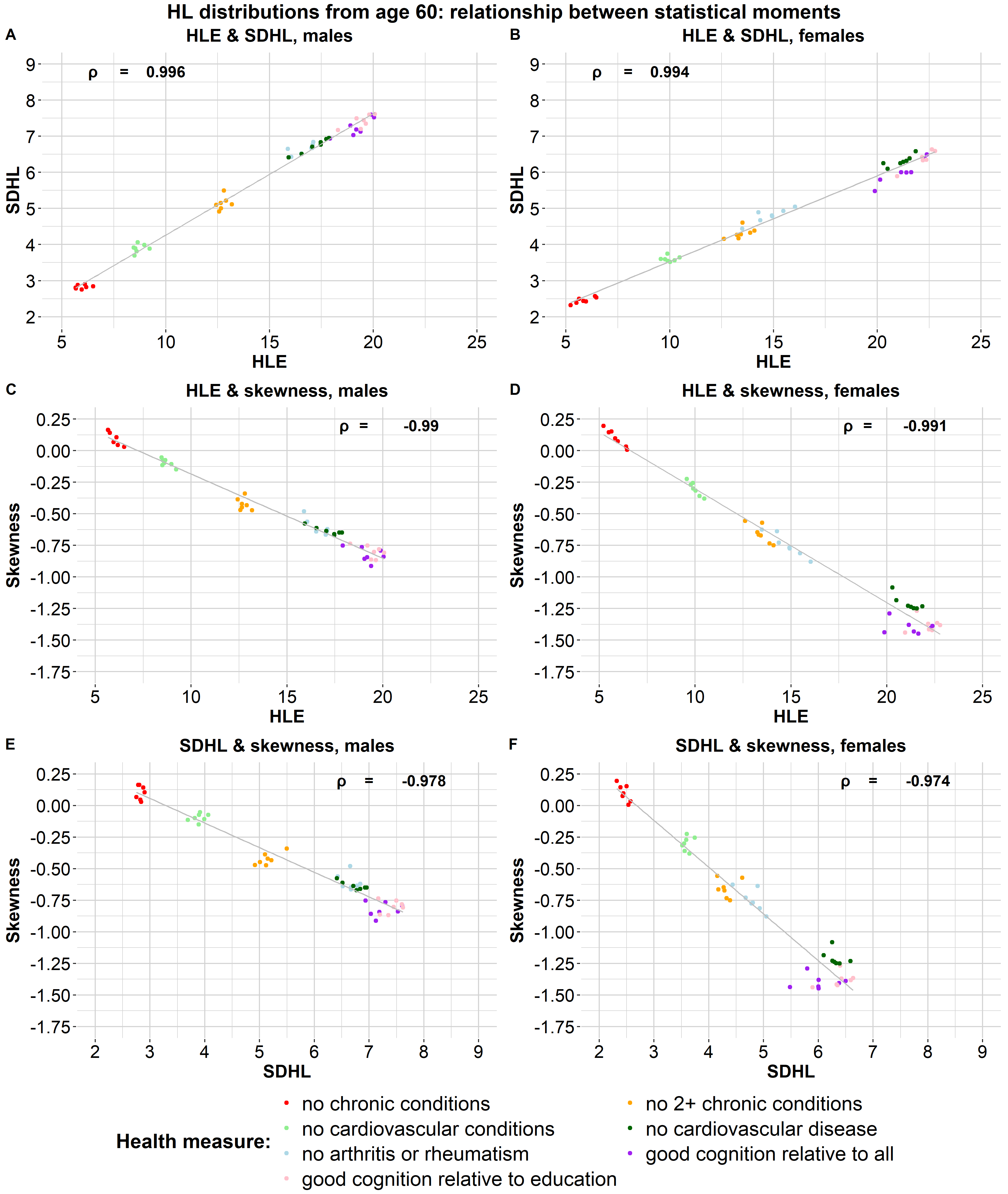
**

**Supplementary Figure S2. Correlations between the statistical moments of healthy longevity distributions.**

Figure notes. Abbreviations: HLE: healthy life expectancy; SDHL: standard deviation of healthy longevity.

$\boldsymbol{\rho}$ refers to the Pearson’s correlation coefficient.

Within each figure panel, there are 7 observations for each health measure, one for each of the seven SHARE waves included in the analysis.

**Supplementary Table S8. Definitions of correlation strength for the current work.**

| **Value of** $\boldsymbol{\rho}$ | **Interpretation of correlation strength** |
| --- | --- |
| >-0.2 & <0 OR >0 & <0.2 | Very weak |
| >-0.4 & <=-0.2 OR >=0.2 & <0.4 | Weak |
| >-0.6 & <=-0.4 OR >=0.4 & <0.6 | Moderate |
| >-0.8 & <=-0.6 OR >=0.6 & <0.8 | Strong |
| >-1 & <=-0.8 OR >=0.8 & <1 | Very strong |


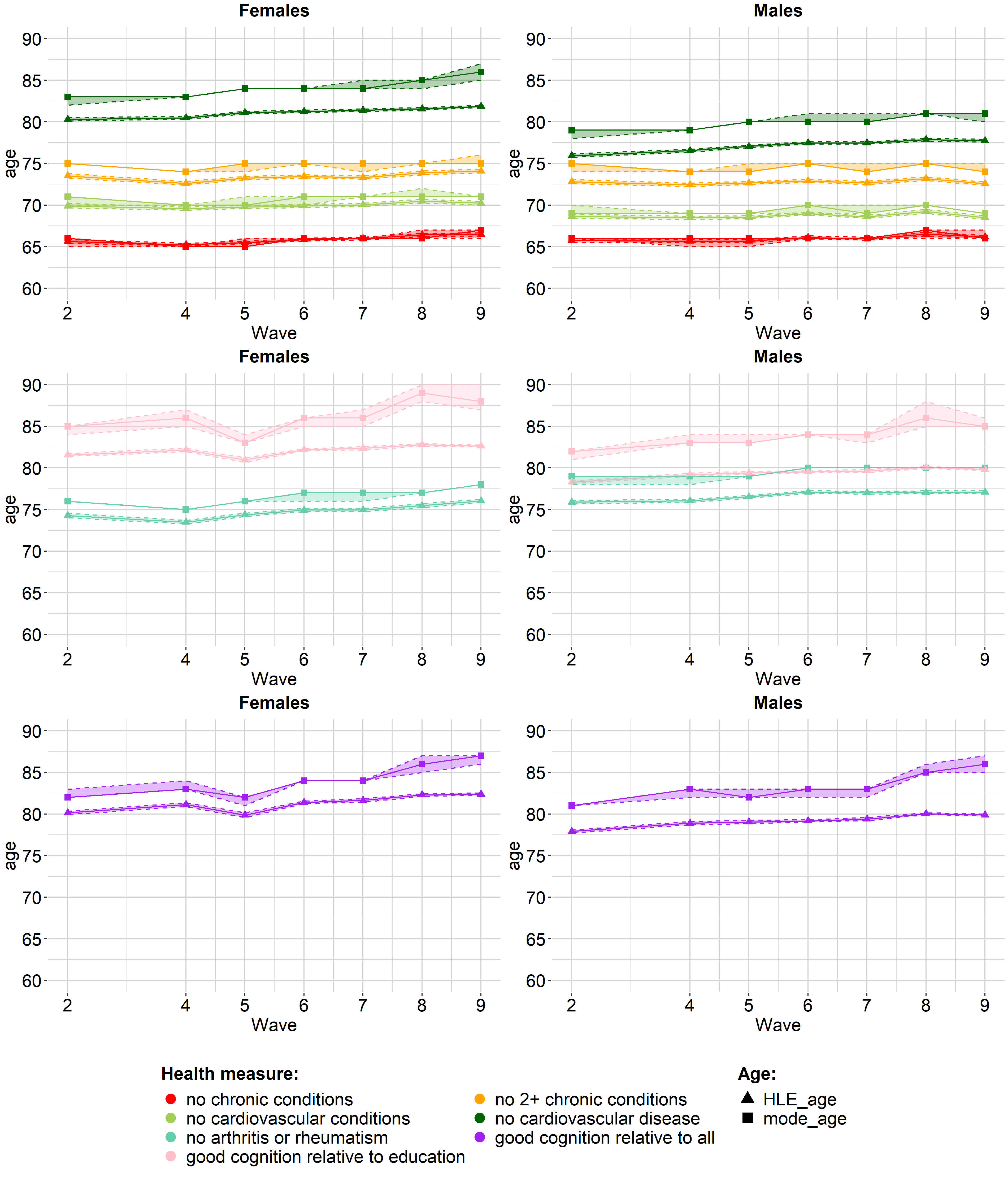


**Supplementary Figure S3. Modal and mean age of health loss.**

**Figure notes.** Abbreviations: HLE: healthy life expectancy.

Symbols (squares or triangles) connected by continuous line: point estimates. Dashed lines: 95% confidence intervals. The confidence intervals for the mode age sometimes correspond the point estimate values: note that in our dataset, mode age was an integer age, while HLE age included decimals.


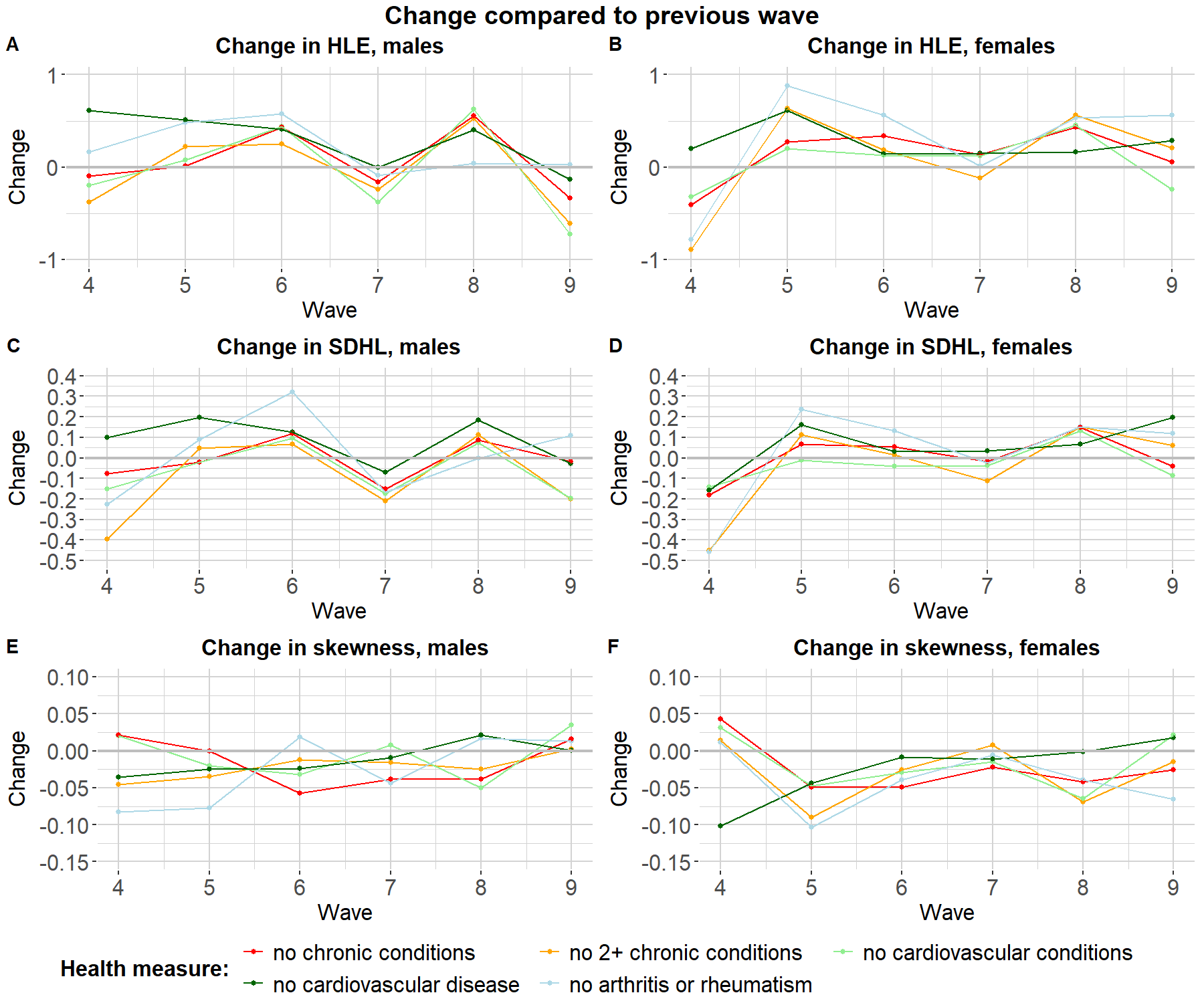


**Supplementary Figure S4. Change in statistical moments compared to the previous wave: health measures related to chronic conditions.**

**Figure notes.** Abbreviations: HLE: healthy life expectancy; SDHL: standard deviation of healthy longevity. When the change value is positive, there has been an increase compared to the previous wave. When the change value is negative, there has been a decrease compared to the previous wave.

Figure based on point estimates.

**
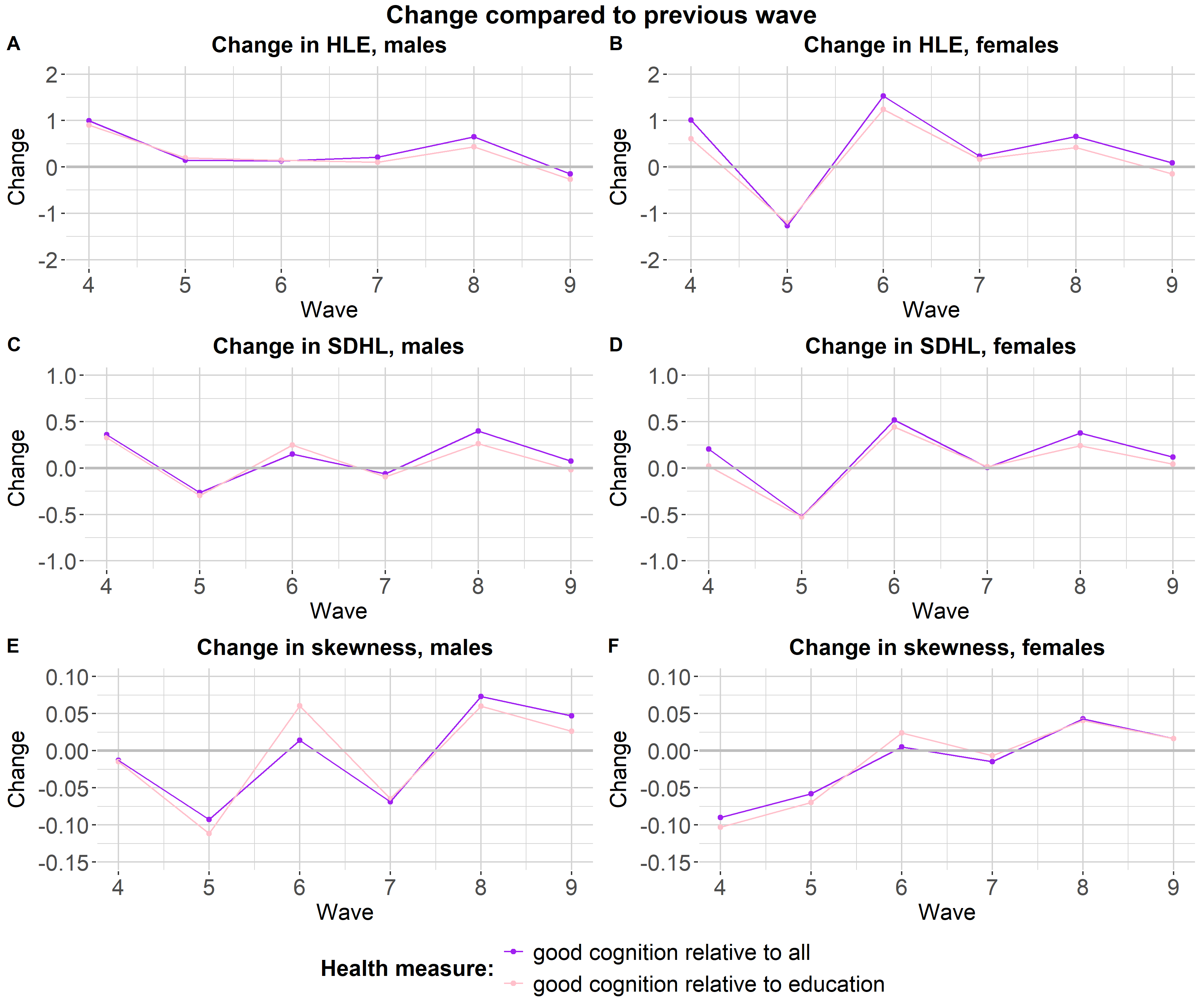
**

**Supplementary Figure S5. Change in statistical moments compared to the previous wave: good cognition measures.**

**Figure notes.** Abbreviations: HLE: healthy life expectancy; SDHL: standard deviation of healthy longevity. When the change value is positive, there has been an increase compared to the previous wave. When the change value is negative, there has been a decrease compared to the previous wave.

Figure based on point estimates.

**
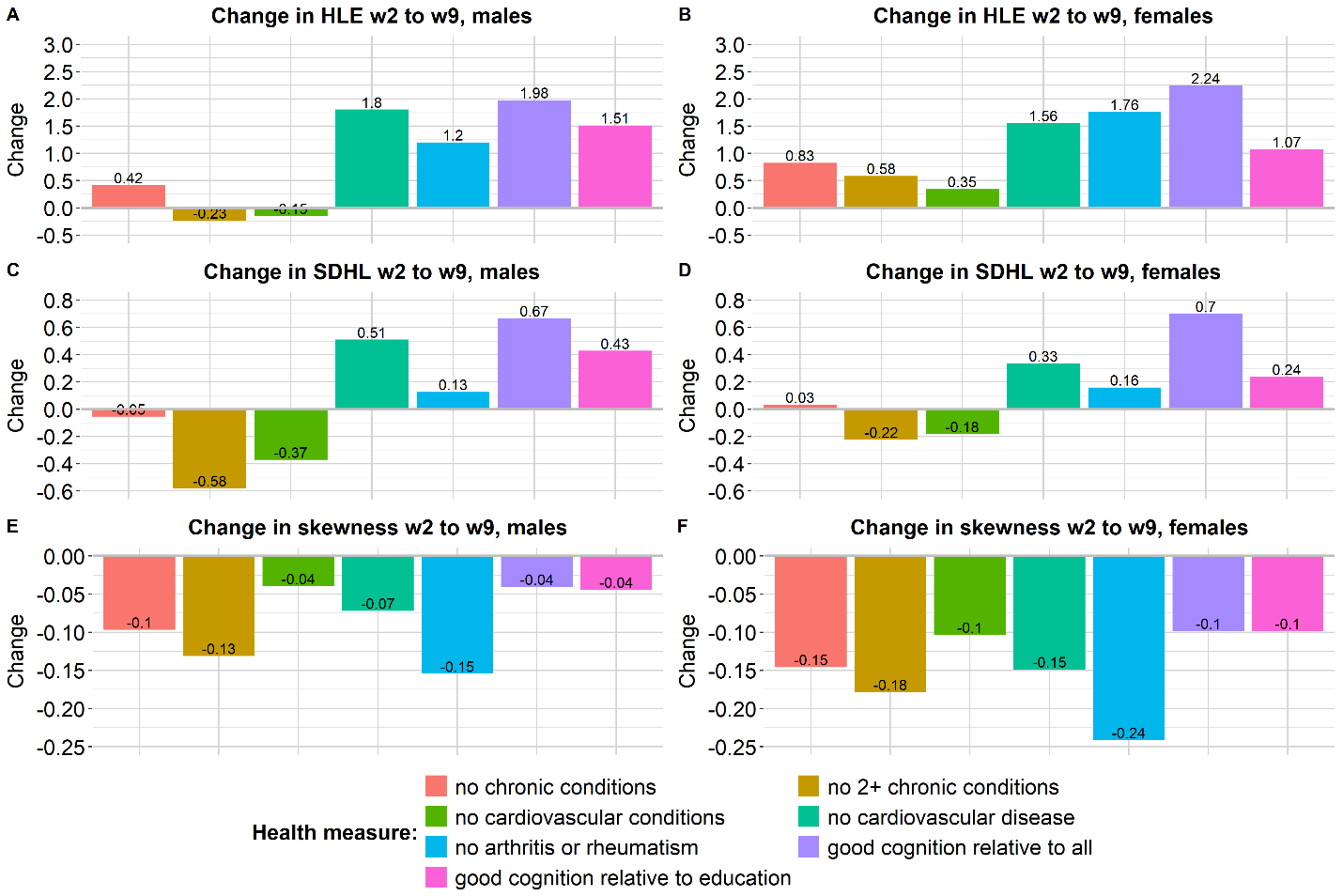
**

**Supplementary Figure S6. Change in HLE, SDHL and skewness from wave 2 to wave 9.**

Figure notes. Abbreviations: HLE: healthy life expectancy; SDHL: standard deviation of healthy longevity; w: wave.

When the change value is positive, there has been an increase from wave 2 to 9. When the change value is negative, there has been a decrease from wave 2 to 9. Figure based on point estimates.

**
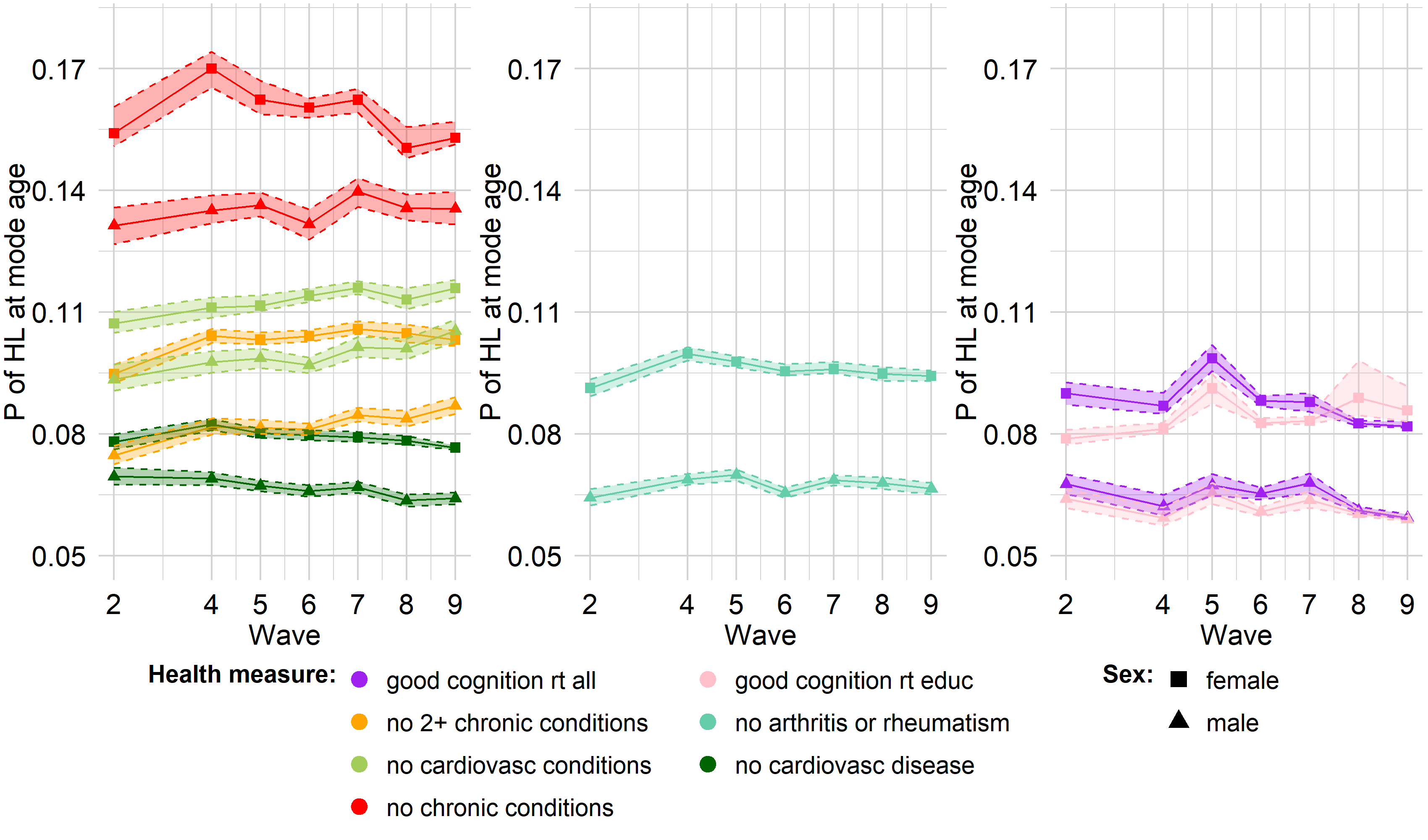
**

**Supplementary Figure S7. Probability of health loss at mode age**

**Figure notes.** Abbreviations: HL: health loss. Symbols (squares or triangles) connected by continuous line: point estimates. Dashed lines: 95% confidence intervals.

**
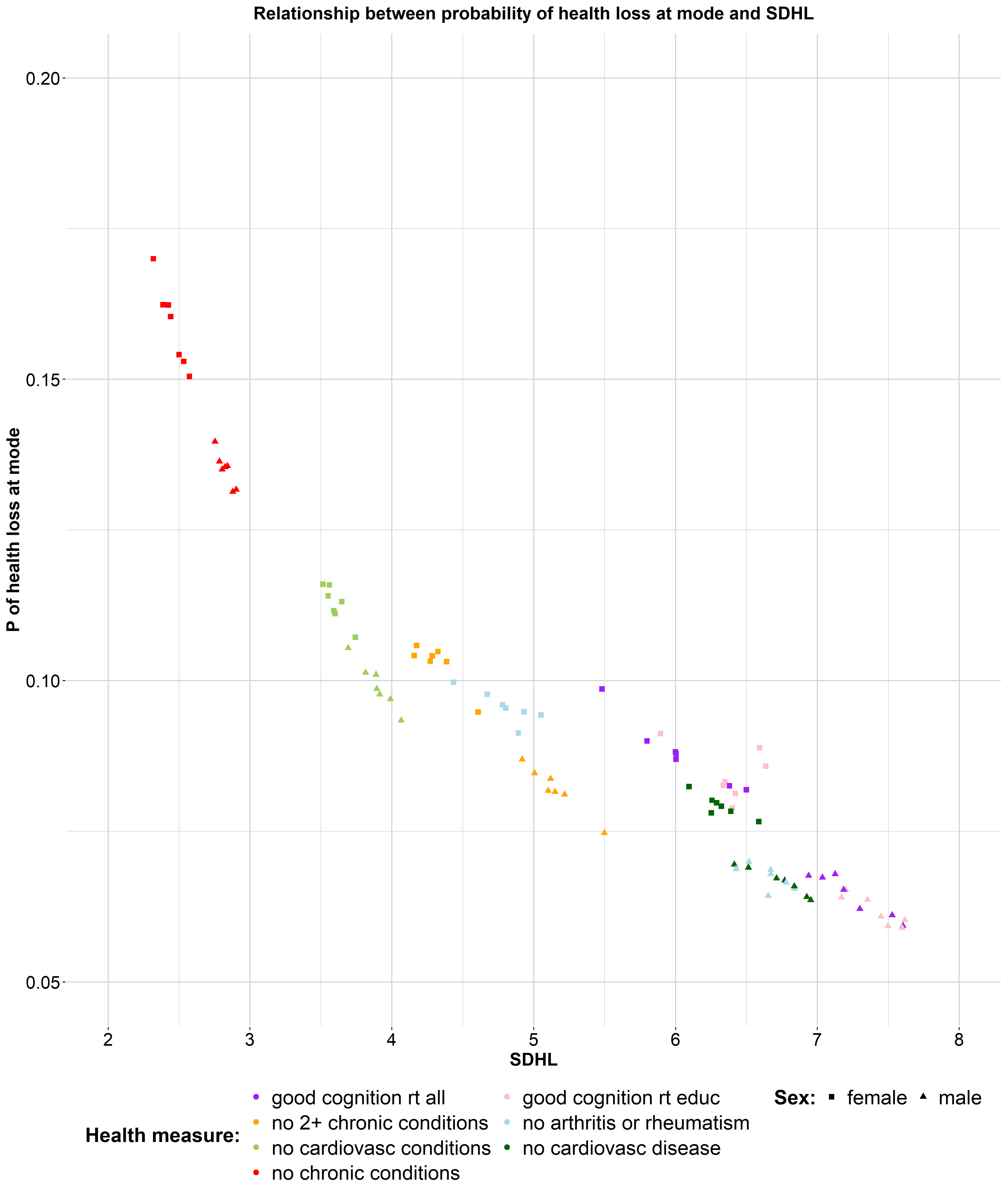
**

**Supplementary Figure S8. Relationship between the probability of health loss at mode and SDHL.**

**Figure notes.** Abbreviations: P: probability; SDHL: standard deviation of healthy longevity.

For each combination of sex and health measure, there are 7 observations, one for each of the 7 included SHARE waves.

Figure based on point estimates only.

**
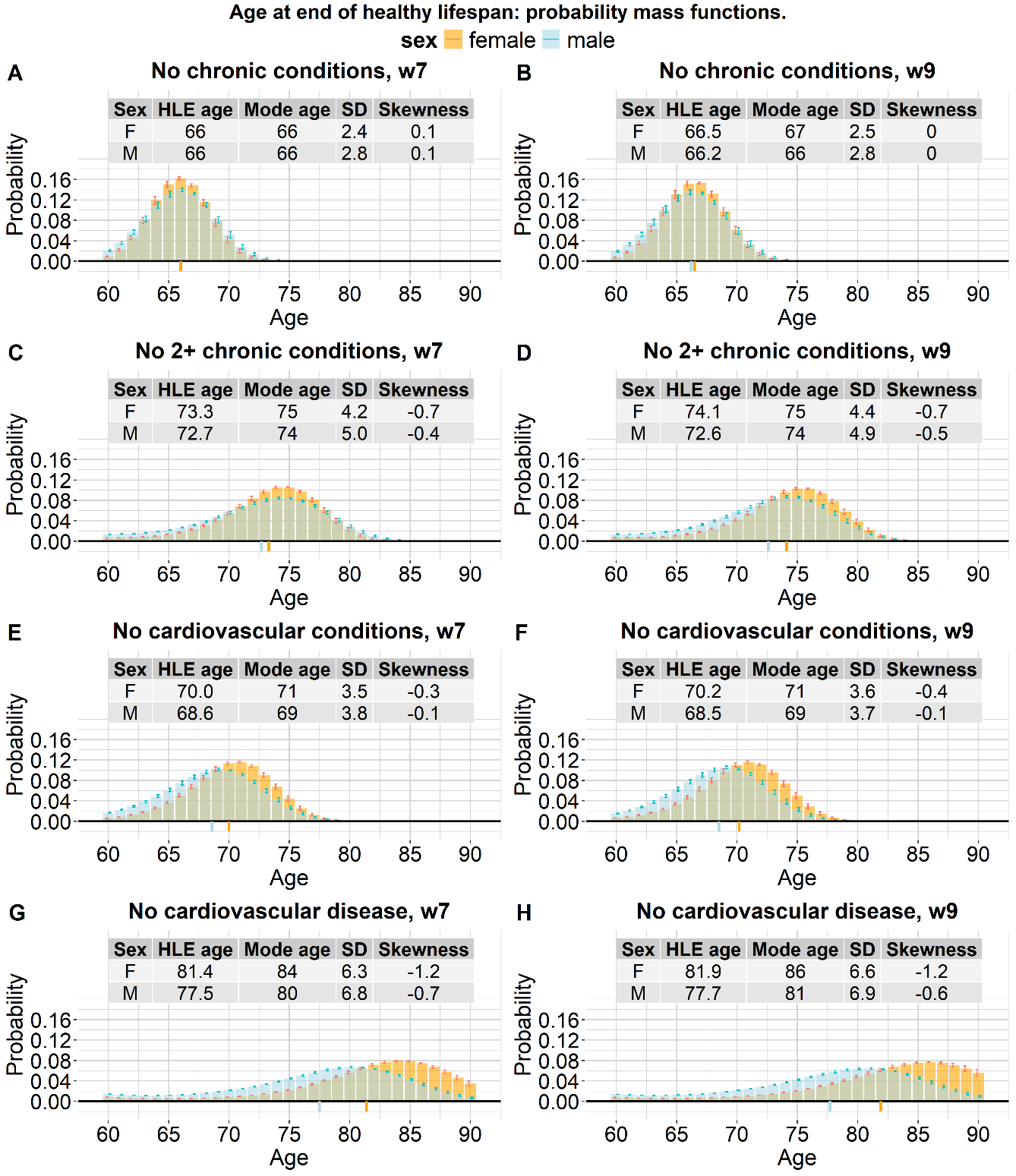
**

**Supplementary Figure S9. Probability mass functions for wave 7 and wave 9, first four health measures.**

**Figure notes.** Abbreviations: F: female; HLE: healthy life expectancy; M: male; SD: standard deviation; w7: wave 7; w9: wave 9.

Wave 7 was conducted in 2017, wave 9 in 2021/22.

The green colour indicates the overlap between the distributions for males and females.

The coloured lines below the x axis show the HLE age for each sex.

The HLE age was equal to 60 + HLE at age 60.

The 95% CIs for the probabilities of health loss are shown, but for simplicity, the reported values are only point estimates.

**
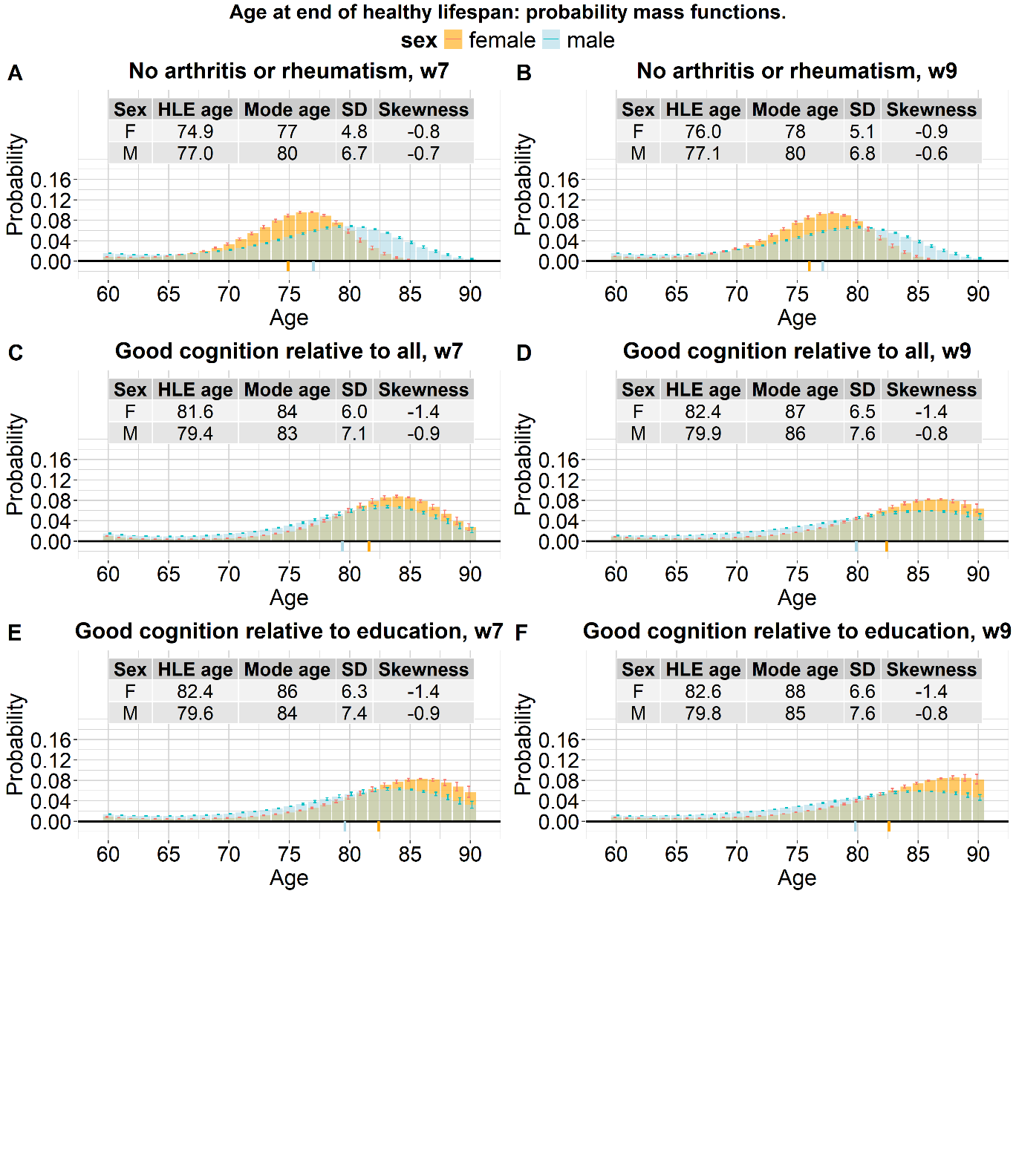
**

**Supplementary Figure S10. Probability mass functions for wave 7 and wave 9, last three health measures.**

**Figure notes.** Abbreviations: F: female; HLE: healthy life expectancy; M: male; SD: standard deviation; w7: wave 7; w9: wave 9.

Wave 7 was conducted in 2017, wave 9 in 2021/22.

The green colour indicates the overlap between the distributions for males and females.

The coloured lines below the x axis show the HLE age for each sex.

The HLE age was equal to 60 + HLE at age 60.

The 95% CIs for the probabilities of health loss are shown, but for simplicity, the reported values are only point estimates.

**
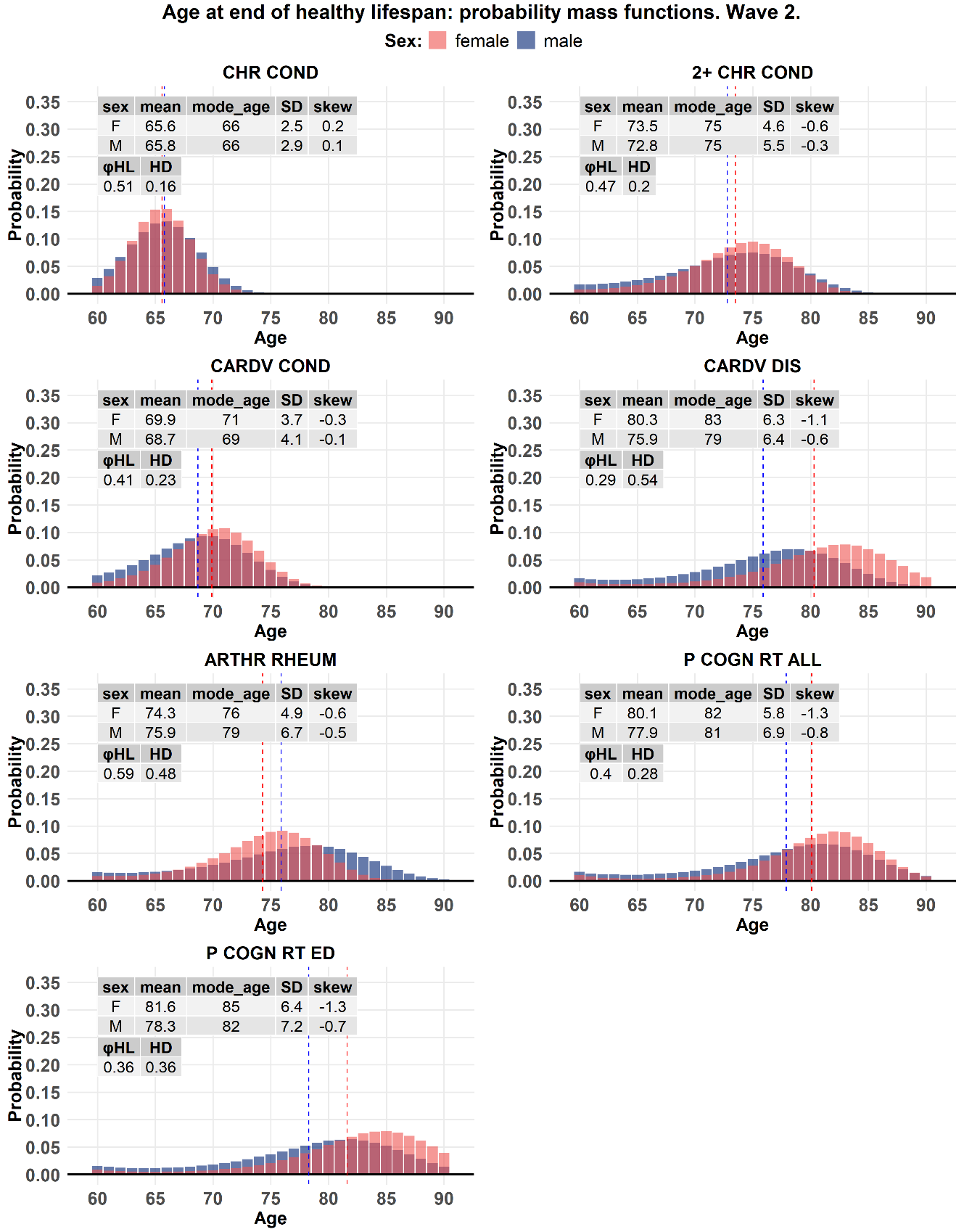
**

**Supplementary Figure S11. Probability mass functions, wave 2, with** $\boldsymbol{\varphi HL}$ **and Hellinger distance.**

**Figure notes.** Abbreviations: arthr rheum: arthritis or rheumatism; cardv cond: cardiovascular conditions; cardv dis: cardiovascular disease; chr cond: chronic conditions; p cogn rt all: poor cognition relative to all person-wave observations; p cogn rt ed: poor cognition relative to the person-wave observations of the same educational level.

Figure based on point estimates only.

The vertical dashed lines indicate the mean values.

**
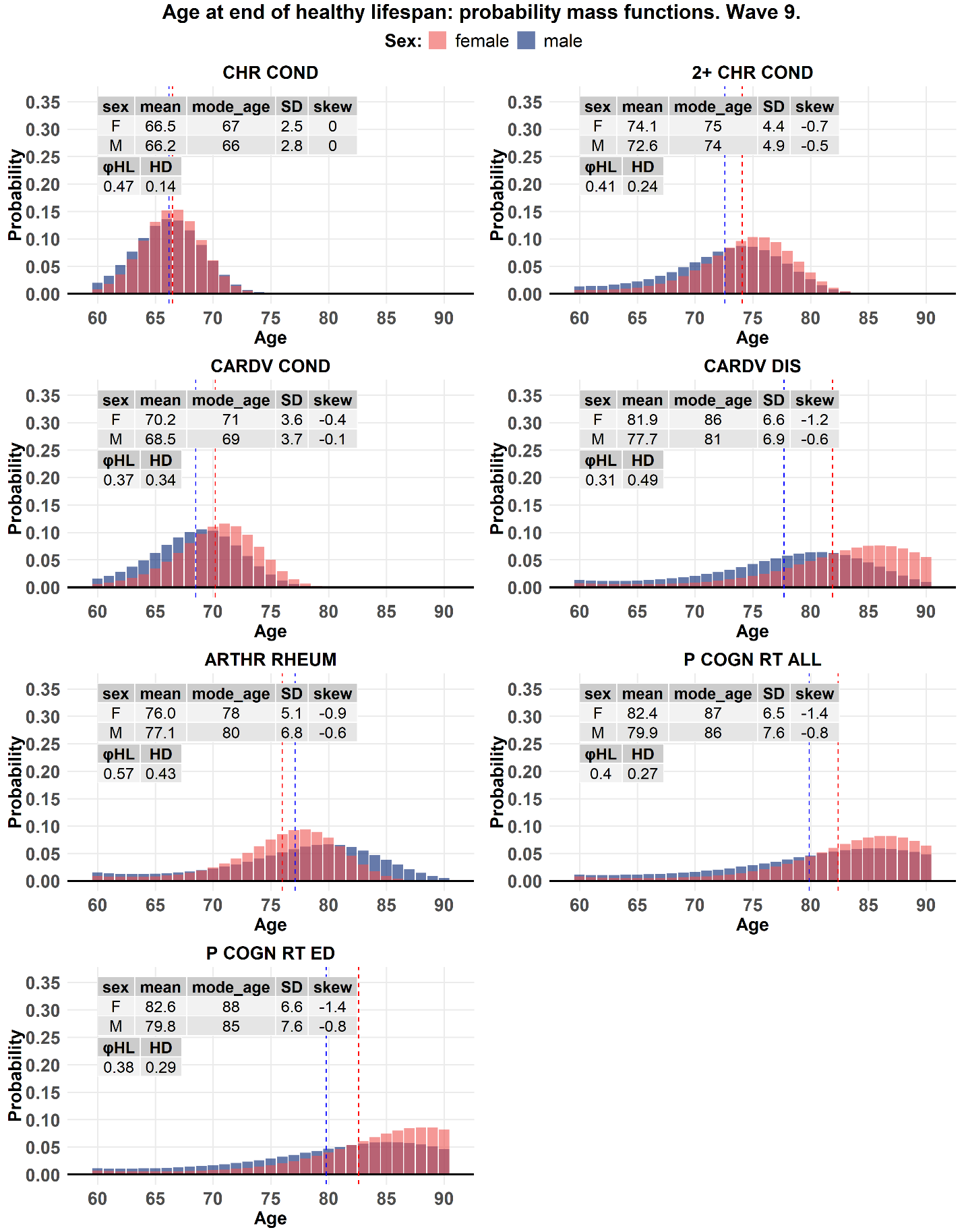
**

**Supplementary Figure S12. Probability mass functions, wave 9, with** $\boldsymbol{\varphi HL}$ **and Hellinger distance.**

**Figure notes.** As per Figure S9.


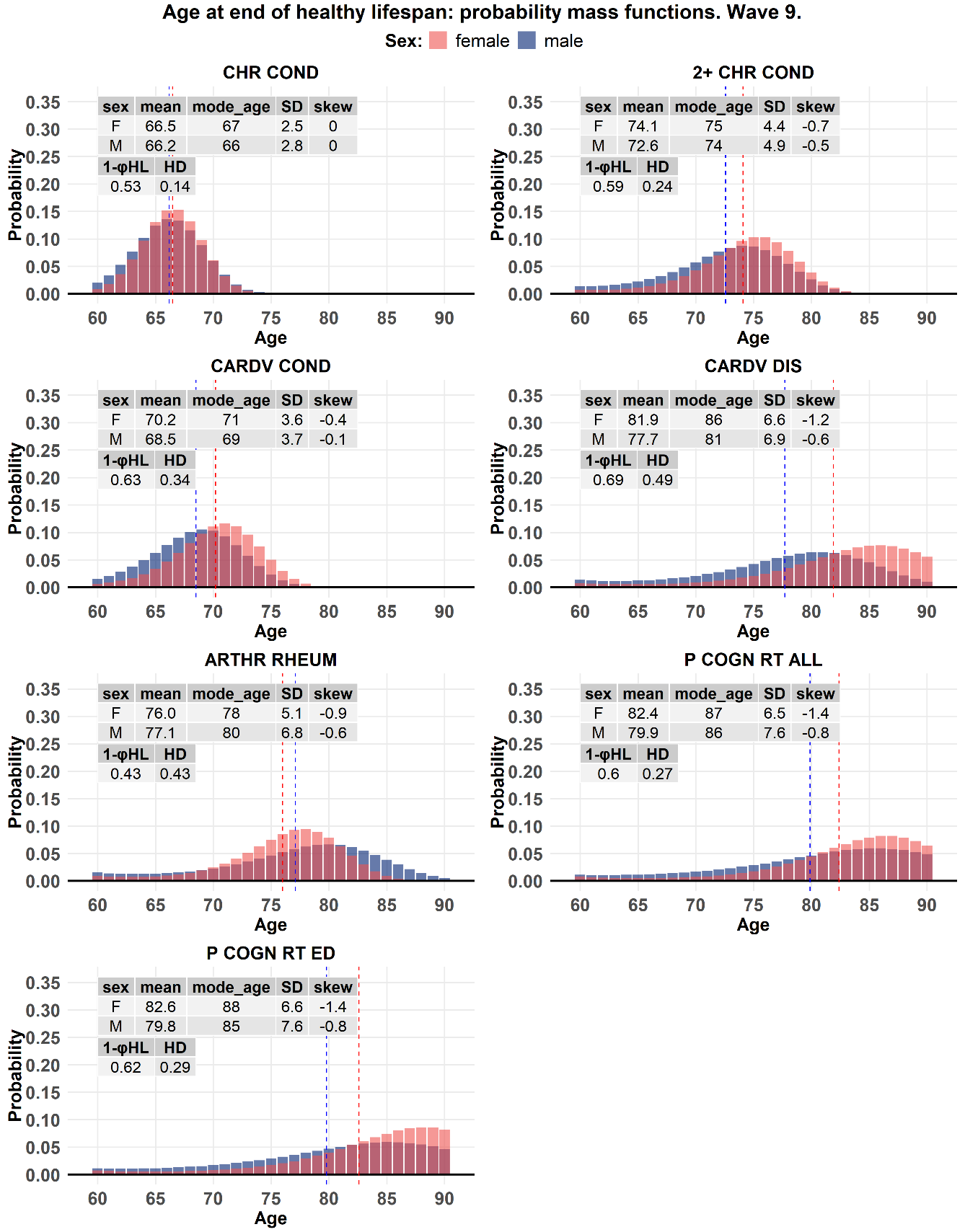


Supplementary Figure S13. Probability mass functions, wave 9, with (1 - $\boldsymbol{\varphi HL}$) and Hellinger distance.

**Figure notes.** As per Figure S9. (1 - $\varphi HL$) expresses the probability for females to have a longer healthy lifespan than males.

**
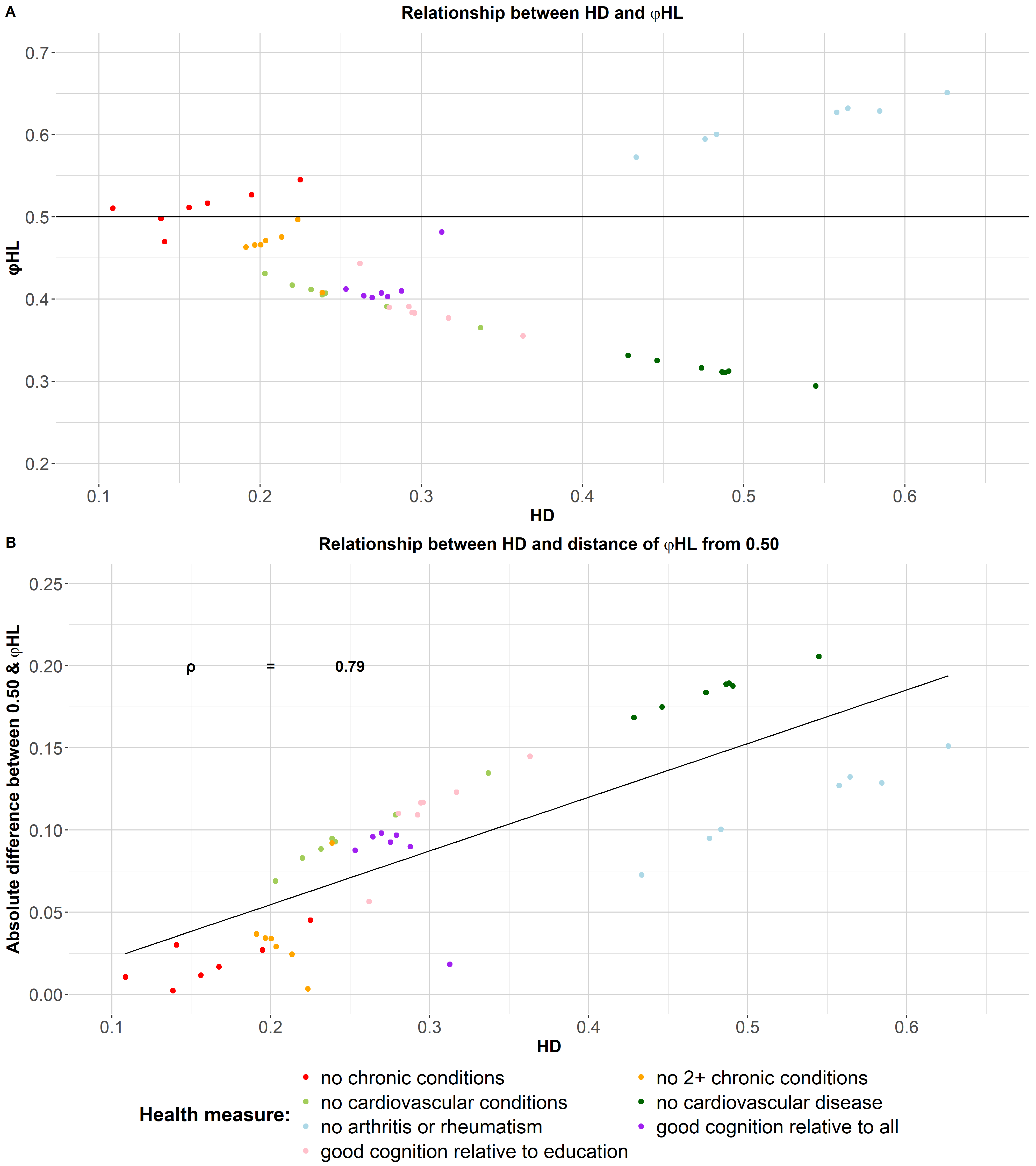
**

Supplementary Figure S14. A. Relationship between Hellinger distance (HD) and $\boldsymbol{\varphi HL}$. B. Correlation between HD and the distance of $\boldsymbol{\varphi HL}$ from 0.50.

Figure notes. Abbreviations: HD: Hellinger distance.

$\boldsymbol{\rho}$ refers to the Pearson’s correlation coefficient.

Figure and correlation coefficient based on point estimates only.

In panel A, the dark grey line corresponds to $\boldsymbol{\varphi HL}$ =0.5. In plot B, the dark grey line corresponds to the least squares model fit.


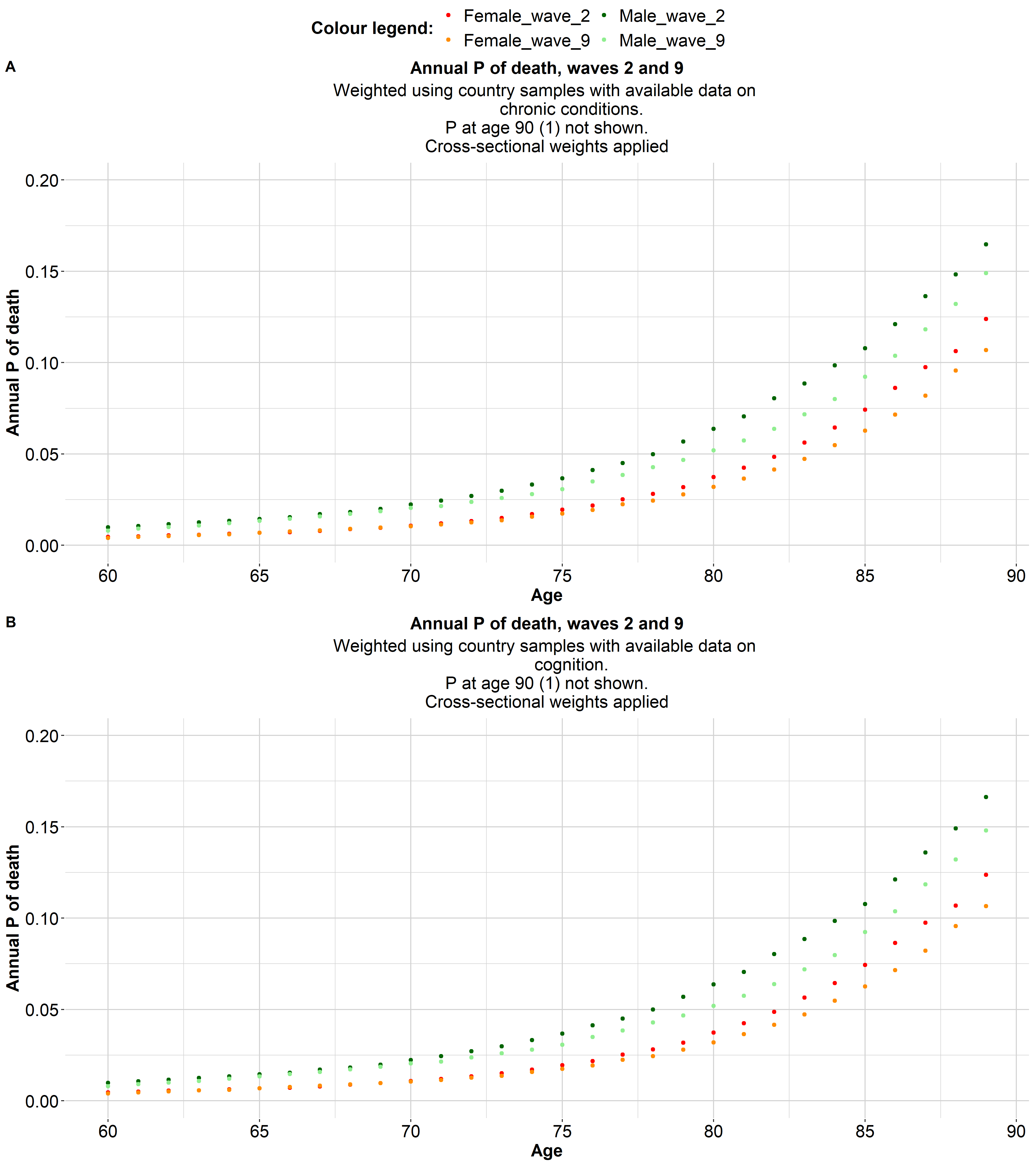


**Supplementary Figure S15. Annual probability of death by age (weighted average across all countries).**

**Figure notes.** Abbreviations: P: probability.

**
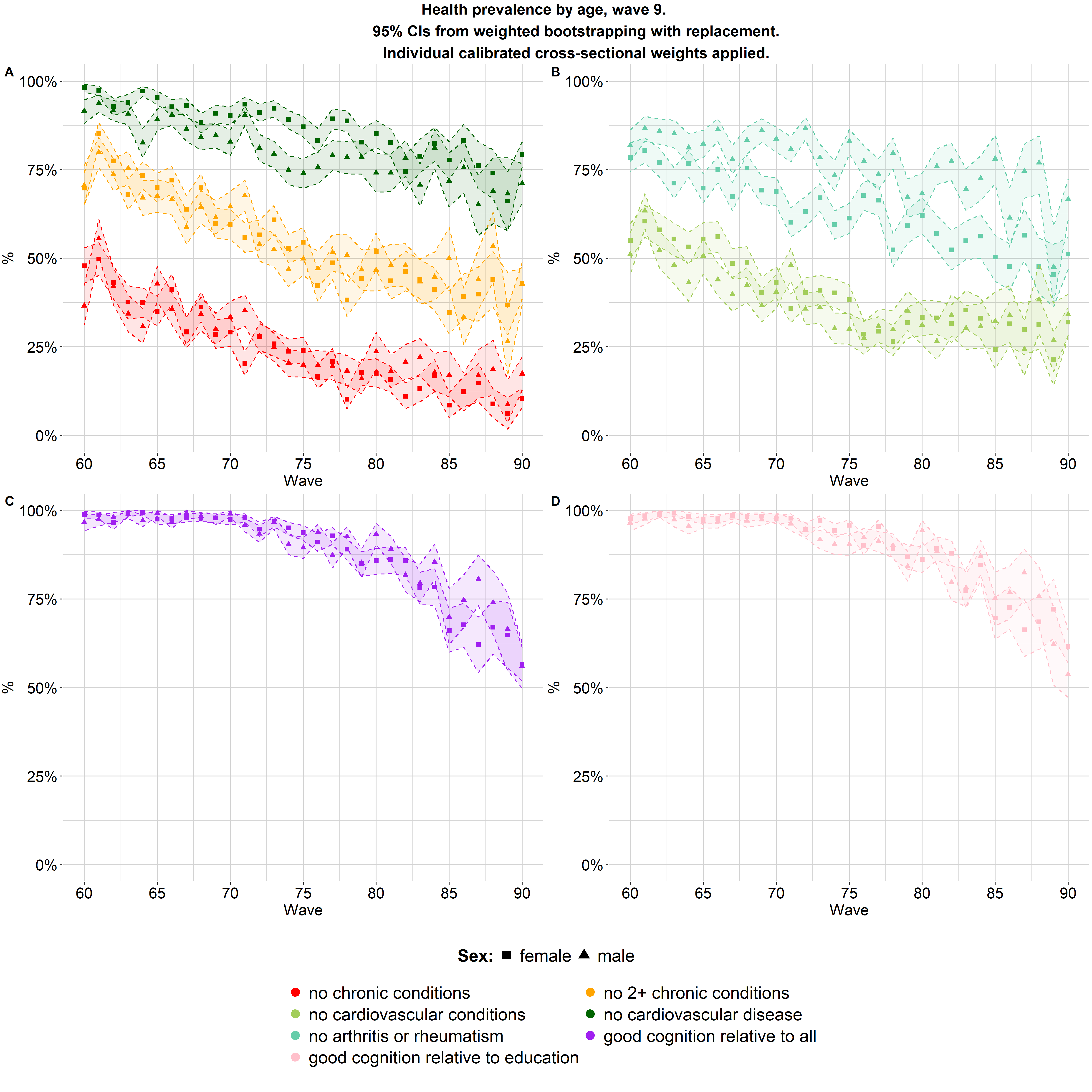
**

**Supplementary Figure S16. Health prevalence with 95% confidence intervals, wave 9.**

**Figure notes.** The figure shows the percentage of people who are “healthy” based on the relevant health measure. 95% CIs based on weighted bootstrapping: 5,000 iterations of resampling with replacement. Individual calibrated cross-sectional weights applied.


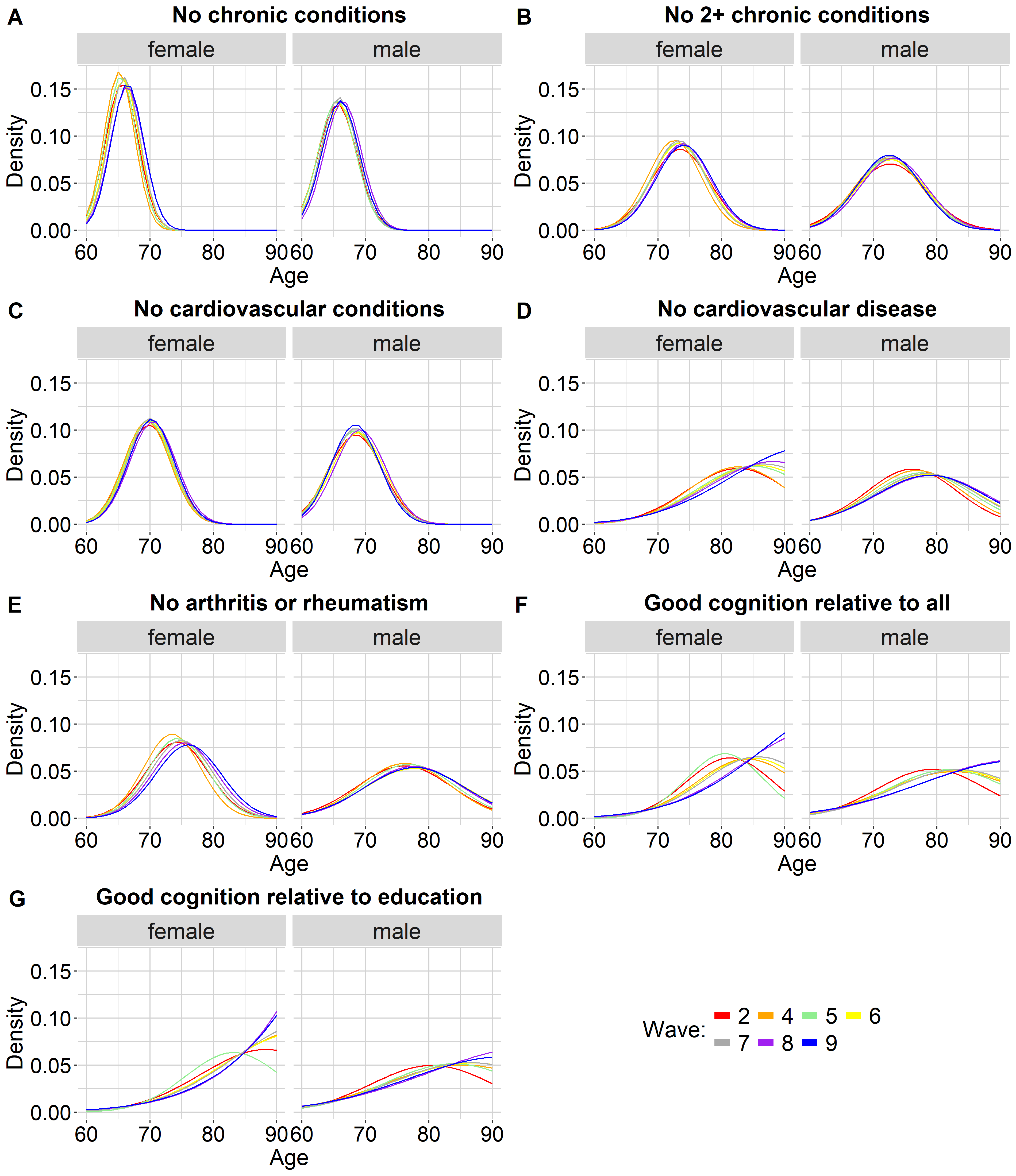


**Supplementary Figure S17. Probability density functions estimated using only the first two statistical moments.**

**Figure notes.** Functions based only on point estimates to simplify the visual representation.

**
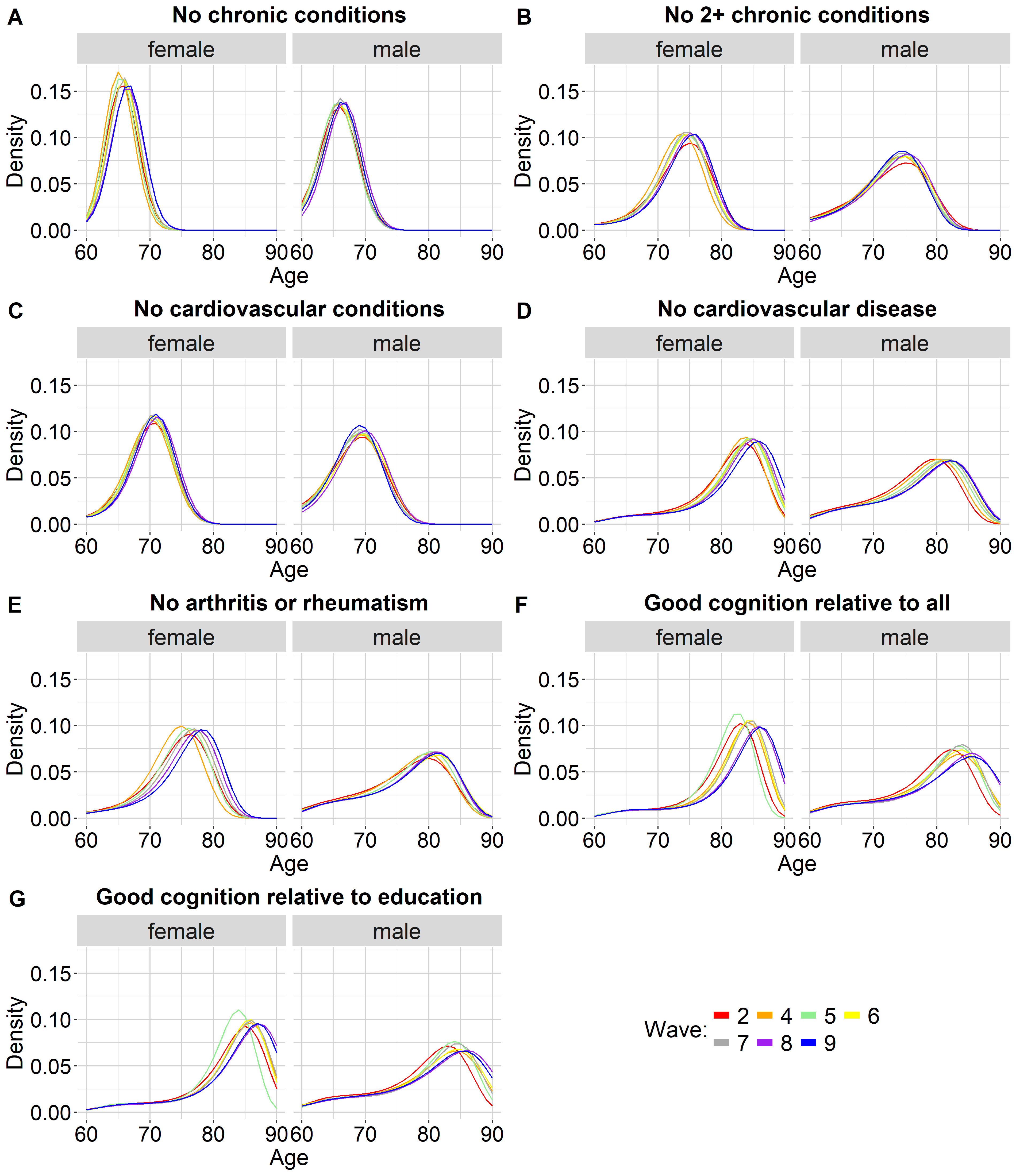
**

**Supplementary Figure S18. Probability density functions estimated using four statistical moments.**

**Figure notes.** Functions based only on point estimates to simplify the visual representation.

**Supplementary Table S9. Validation check on the ages when >=99% cumulative probabilities of health loss were reached based on the maximum entropy method, wave 9.**

| **Health measure** | **Sex** | **Estimate from the maximum entropy method** | **Estimates from the HMD and SHARE data: point estimates (95% CI)** | | |
| --- | --- | --- | --- | --- | --- |
|  |  | **Age when the >=99% cumulative probability of health loss was reached: point estimate (95% CI)** | **Probability of being alive**^a^ **at that age** | **Prevalence of health (i.e., of being free from the condition of interest) at that age** | **Probability of being alive and healthy based on the point estimates**^b^ |
| No chronic conditions | female | 72 (72 to 72) | 0.92 | 0.28 (0.24 to 0.32) | 0.26 |
| No chronic conditions | male | 73 (72 to 73) | Age 72: 0.84  Age 73: 0.82 | Age 72: 0.28 (0.24 to 0.32)  Age 73: 0.25 (0.21 to 0.29) | Age 73: 0.21 |
| No 2+ chronic conditions | female | 82 (82 to 82) | 0.73 | 0.46 (0.41 to 0.52) | 0.34 |
| No 2+ chronic conditions | male | 82 (82 to 82) | 0.57 | 0.48 (0.42 to 0.54) | 0.27 |
| No cardiovascular conditions | female | 77 (77 to 78) | Age 77: 0.85  Age 78: 0.83 | Age 77: 0.29 (0.25 to 0.33)  Age 78: 0.27 (0.23 to 0.31) | Age 77: 0.25 |
| No cardiovascular conditions | male | 76 (76 to 77) | Age 76: 0.75  Age 77: 0.73 | Age 76: 0.27 ( 0.23 to 0.32)  Age 77: 0.31 (0.26 to 0.36) | Age 76: 0.20 |
| No cardiovascular disease | female | 90 (90 to 90) | Not relevant | Not relevant | Not relevant |
| No cardiovascular disease | male | 89 (89 to 90) | Age 89: 0.28 | Age 89: 0.68 (0.58 to 0.78) | 0.19 |
| No arthritis or rheumatism | female | 85 (85 to 85) | 0.63 | 0.50 (0.43 to 0.57) | 0.32 |
| No arthritis or rheumatism | male | 89 (89 to 89) | 0.28 | 0.48 (0.37 to 0.59) | 0.13 |
| Good cognition relative to all | female | 90 (90 to 90) | Not relevant | Not relevant | Not relevant |
| Good cognition relative to all | male | 90 (90 to 90) | Not relevant | Not relevant | Not relevant |
| Good cognition relative to educ | female | 90 (90 to 90) | Not relevant | Not relevant | Not relevant |
| Good cognition relative to educ | male | 90 (90 to 90) | Not relevant | Not relevant | Not relevant |

**Table notes.** Abbreviations: CI: confidence interval. ^a^ The probability of being alive at a specific age was calculated as follows: (1-p of death at age 60)* (1-p of death at age 61)*(1-p of death at age 62)… and so on until the p of death at the age prior to the age of interest. Where p stands for probability.

^b^ Calculated by multiplying the p of being alive at age x by the p of health prevalence at the same age.

# References

1. SHARE. SHARE w2 questionnaire version 2.7 2006-09-21 [Internet]. 2006 [cited 2025 Mar 15]. https://share-eric.eu/fileadmin/user_upload/Questionnaires/Q-Wave_2/w2_en_capi_main-Generic.pdf. Accessed 15 Mar 2025

2. SHARE. Main questionnaire. Wave 4. [Internet]. n.d. [cited 2025 Mar 15]. https://share-eric.eu/fileadmin/user_upload/Questionnaires/Q-Wave_4/w4_en_capi_main-Generic.pdf. Accessed 15 Mar 2025

3. SHARE. Main questionnaire & end of life questionnaire. Wave 5 [Internet]. n.d. [cited 2025 Mar 15]. https://share-eric.eu/fileadmin/user_upload/Questionnaires/Q-Wave_5/w5_en_capi_main-Generic.pdf. Accessed 15 Mar 2025

4. SHARE. Main questionnaire & end of life questionnaire. Wave 6 [Internet]. n.d. [cited 2025 Mar 15]. https://share-eric.eu/fileadmin/user_upload/Questionnaires/Q-Wave_6/w6_en_capi_main-Generic.pdf. Accessed 15 Mar 2025

5. SHARE. Main questionnaire. Wave 7. [Internet]. n.d. [cited 2025 Mar 15]. https://share-eric.eu/fileadmin/user_upload/Questionnaires/Q-Wave_7/w7_en_capi_main-Generic.pdf. Accessed 15 Mar 2025

6. SHARE. Main questionnaire. Wave 8. [Internet]. n.d. [cited 2025 Mar 15]. https://share-eric.eu/fileadmin/user_upload/Questionnaires/Q-Wave_8/paperverstion_en_GB_8_2_5b.pdf. Accessed 15 Mar 2025

7. SHARE. Main questionnaire. Wave 9. [Internet]. n.d. [cited 2025 Mar 15]. https://share-eric.eu/fileadmin/user_upload/Questionnaires/Q-Wave_9/paperversion_en_GB_9_2_2a.pdf. Accessed 15 Mar 2025

8. Zheng F, Yan L, Yang Z, Zhong B, Xie W. HbA1c, diabetes and cognitive decline: the English Longitudinal Study of Ageing. Diabetologia. 2018;61:839–48. https://doi.org/10.1007/s00125-017-4541-7

9. Han FF, Wang HX, Wu JJ, Yao W, Hao CF, Pei JJ. Depressive symptoms and cognitive impairment: A 10-year follow-up study from the Survey of Health, Ageing and Retirement in Europe. Eur Psychiatry. 2021;64:e55. https://doi.org/10.1192/j.eurpsy.2021.2230

10. SHARE. Survey of Health, Ageing and Retirement in Europe (SHARE) Release Guide 9.0.0 [Internet]. 2024 [cited 2025 Jan 15]. https://share-eric.eu/fileadmin/user_upload/Release_Guides/SHARE_release_guide_9-0-0.pdf. Accessed 15 Jan 2025
